# Severe dengue in children associates with dysregulation of lipid homeostasis, complement cascade and retinol transport

**DOI:** 10.1002/ctm2.1271

**Published:** 2023-06-01

**Authors:** Aleksha Panwar, Rinki Kumar, Renu Goel, Suruchi Aggarwal, Shweta Saraswat, Priyanka Bansal, Zaozianlungliu Gonmei, Gurudyal Toteja, Amit Yadav, Rakesh Lodha, Nirpendra Singh, Guruprasad Medigeshi

**Affiliations:** 1 Clinical and Cellular Virology Laboratory, Infection and Immunology Division, Translational Health Science and Technology Institute, Faridabad, Haryana, India; 2 Computational andMathematical Biology Centre, Translational Health Science and Technology Institute, Faridabad, Haryana, India; 3 Department of Infection and Immunity, Translational Health Science and Technology Institute, NCR Biotech Cluster, Faridabad, Haryana, India; 4 Centre for Promotion of Nutrition Research and Training with Special Focus on North East, Indian Council of Medical Research, Tribal & Inaccessible Population, New Delhi, India; 5 Department of Pediatrics, All India Institute of Medical Sciences, New Delhi, India; 6 inStem, Bangalore, Karnataka, India

Dear Editor,

Dengue is the most prevalent mosquito-borne flavivirus infection in humans with 3.9 billion people at risk of infection and 70% of the disease burden is in Asia.^1,2^ The four serologically and genetically distinct Dengue viruses (DENV1-4) can cause mild flu-like symptoms to life-threatening disease involving vascular leakage and thrombocytopenia which can lead to shock and organ failure. Our study was designed to identify biomarkers of severe dengue by quantitative proteomics based on isobaric Tag for Relative and Absolute Quantitation (iTRAQ) labelling of plasma samples obtained from four different disease severity conditions: convalescent (CONV; *n* = 5), mild dengue/dengue illness (DI; *n* = 5), severe dengue without fluid leak (SD No FL; *n* = 5) and severe dengue with fluid leak (SD with FL; *n* = 5). The samples were divided into five sets for 4-plex iTRAQ labelling with each set consisting of all the four disease conditions (Figure S1 and Table S1). The distribution of peptide counts obtained across proteins that are differentially regulated in dengue infection is shown (Figure S2). Ninety-six proteins identified in DI, 101 in SD and 104 in SD with FL, respectively, are represented using Venn diagram to identify proteins unique to each of the three clinical symptoms (Figure 1A). We selected 54 proteins, which were identified in at least two out of the five iTRAQ runs (Dataset S1) and the expression levels of these proteins in dengue samples relative to convalescent samples were depicted in a heat map (Figure 1B). Network analysis was performed by Search Tool for the Retrieval of Interacting Genes/Proteins to predict interactions (both direct and indirect/functional) among the differentially expressed proteins (Figure 1C). A subset of identified proteins clustered into two prominent functional network namely the plasma lipid transport and complement pathways. Analysis for biological enrichment, molecular function and Kyoto encyclopaedia of Genes and Genomes pathway analysis also identified plasma lipoprotein regulation and complement activation as the two major pathways represented by the cluster of proteins (Dataset S2). Pathway analysis by reactome identified platelet degranulation, complement cascade and plasma lipoprotein homeostasis as the top 10 pathways after false-discovery rate correction (Dataset S3 and Figures S3-S5). Venn diagram shows the distribution of 54 proteins among the three dengue groups (Figure 2A). We identified 11 proteins out of 54 that showed overall significant up- or downregulation (< 0.5- or > 1.5-fold) (Figure 2B) in dengue group relative convalescent samples. We next analysed the difference in expression levels relative to SD (no FL) in all the 54 proteins (Figure 2C-S). Out of the 11 proteins that showed overall dysregulation in dengue, only Apolipoprotein E (APOE) (Figure 2E), Haptoglobin-related protein (HPTR) (Figure 2K), Galectin-3-binding protein (LG3BP) (Figure 2M) showed further differences relative to SD (No FL) samples. We found that plasma protease C1 inhibitor (IC1 or SERPING1) (Figure 2C), CD14 (Figure 2F), leucine-rich alpha-2-glycoprotein (A2GL or LRG1) (Figure 2H), Complement component C2 and C9 (Figure 2I and L) were all uniformly upregulated across all the three disease symptoms. Apolipoprotein C1 (APOC1) (Figure 2D) and Histidine-rich glycoprotein (HRG) (Figure 2J) was downregulated in all the three conditions relative to convalescent samples (Figure 2L). The levels of retinol-binding protein (RET4) was lowest in SD with FL samples although not statistically significant (Figure 2G). In addition, we found upregulation of acute phase apolipoproteins Serum Amyloid A1 and A2 (SAA1 and SAA2) in SD (No FL) samples (Figure 2N and O). Ficolin-3 (FCN3), which activates the lectin pathway of the complement cascade, was significantly downregulated in SD with fluid leak samples (Figure 2P). Heparin Cofactor 2 (HEP2), an inhibitor of thrombin, levels were lower in SD (No FL) samples (Figure 2Q). Both lipopolysaccharide-binding protein (LBP) and C-reactive protein (CRP) levels were elevated in SD (with or without FL) samples (Figure 2R and S) suggesting an exaggerated inflammatory response.

A subset of proteins from Figure 2 were validated by high resolution multireaction monitoring (HR-MRM) based on tryptic peptides, which typically range from 5 to 25 amino acids in length and usually form multiple, charged, positive ions by MS/MS fragmentation (Table S2). We used an independent set of clinical samples from convalescent (*n* = 5), DI (*n* = 5) and SD with FL (*n* = 5) for this purpose (Figure S6). We observed a significant upregulation of the peptide VEIFYR of LG3BP in HR-MRM in SD samples relative to DI samples (Figure 3A). The coelution profile of all the transitions for this peptide is shown (Figure S7). The level of HEP2 peptide was lower in mild and severe dengue; however, it was not statistical significant due to huge variation in CONV samples (Figure 3B). HRG peptide was significantly downregulated in SD cases by HR-MRM (Figure 3C). The peptide from IC1/SERPING1 was present at significantly higher levels in SD cases (Figure 3D). The parent ion of selected peptide (FICPLTGLWPINTLK) from protein APOH and APOA4 (ALVQQMEQLR and SLAELGGHLDQQVEEFR) showed downregulation in both DI and SD (Figures 3E and F and S8 and S9). The peptide ‘VAAGAFQGLR’ from LRG1 showed no significant difference between the three conditions (Figures 3G and S10) which was also confirmed in clinical samples from DI patients (*n* = 12) or SD (*n* = 15) or from other febrile illness (OFI) (*n* = 6) (Figure S11). The two RET4 peptides FSGTWYAMAK and YWGVASFLQK showed significant downregulation in SD with FL samples in HR-MRM (Figures 3H and S12). RET4 is secreted mainly by the liver tissue and is involved in retinol transport in blood plasma. It is known that DHF induces increased retinoic acid receptor activation which leads to inhibition of production and secretion of RET4.^3^ We measured the retinol levels in serum samples from DI and SD and in convalescent samples as retinol is bound to RET4 in a 1:1 ratio.^4^ We observed significantly lower levels of retinol in SD relative to convalescent samples (Figure 3I) further correlating with reduced RET4 levels indicating Vitamin A deficiency in dengue infection. Lower levels of total cholesterol, high-density lipoproteins and low-density lipoproteins in plasma were associated with severe dengue infection.^5–7^ We next validated the modulation of human apolipoprotein levels in serum samples of healthy control (*n* = 8), patients with other febrile illness (OFI) (*n* = 5), DI (*n* = 12), and SD (*n* = 15) using multiplex (11-plex) bead-based assay panel. The assay included detection and quantitation of Apo-AI, Apo-AII, Apo-B100, Apo-CII, Apo-CIII, Apo-D, Apo-E, Apo-E4, Apo-H, Apo-J and Apo-M. The levels of Apo-AII and Apo-B100 were beyond the detection limit of the assay and were not considered for final analysis. Seven apolipoproteins namely Apo-AI, Apo-CII, Apo-CIII, Apo-D, Apo-H, Apo-J and Apo-M levels were significantly reduced in SD samples as compared to DI (Figure 4A-D and G-I) whereas Apo-E and Apo-E4 showed no significant difference between DI and SD (Figure 4E and F).

Severe dengue is primarily a clinical outcome of dysregulation of immune response and is a multifactorial event involving both innate and adaptive arms of the immune response.^8–10^ We have identified differential expression of proteins regulating lipid homeostasis (HPTR, SAA1, SAA2 and apolipoproteins), complement activation (IC1, FCN3), coagulation cascade (HEP2, HRG) and retinol transport (RET4) pathways in severe dengue. Our results provide a basis for further validation of these proteins in larger cohorts to support their potential as a biomarker of severe dengue, which may also help in developing interventions for severe dengue. Please see supplementary discussion for further details.

## Supplementary Material

Additional supporting information can be found online in the Supporting Information section at the end of this article.

Supplementary dataset 1

Supplementary dataset 2

Supplementary Discussion

Supplementary Figure Legends

Supplementary Figure S1

Supplementary Figure S2

Supplementary Figure S3

Supplementary Figure S4

Supplementary Figure S5

Supplementary Figure S6

Supplementary Figure S7

Supplementary Figure S8

Supplementary Figure S10

Supplementary Figure S11

Supplementary Figure S12

Supplementary Methods

Supplementary Table S1 and S2

## Figures and Tables

**Figure 1:**
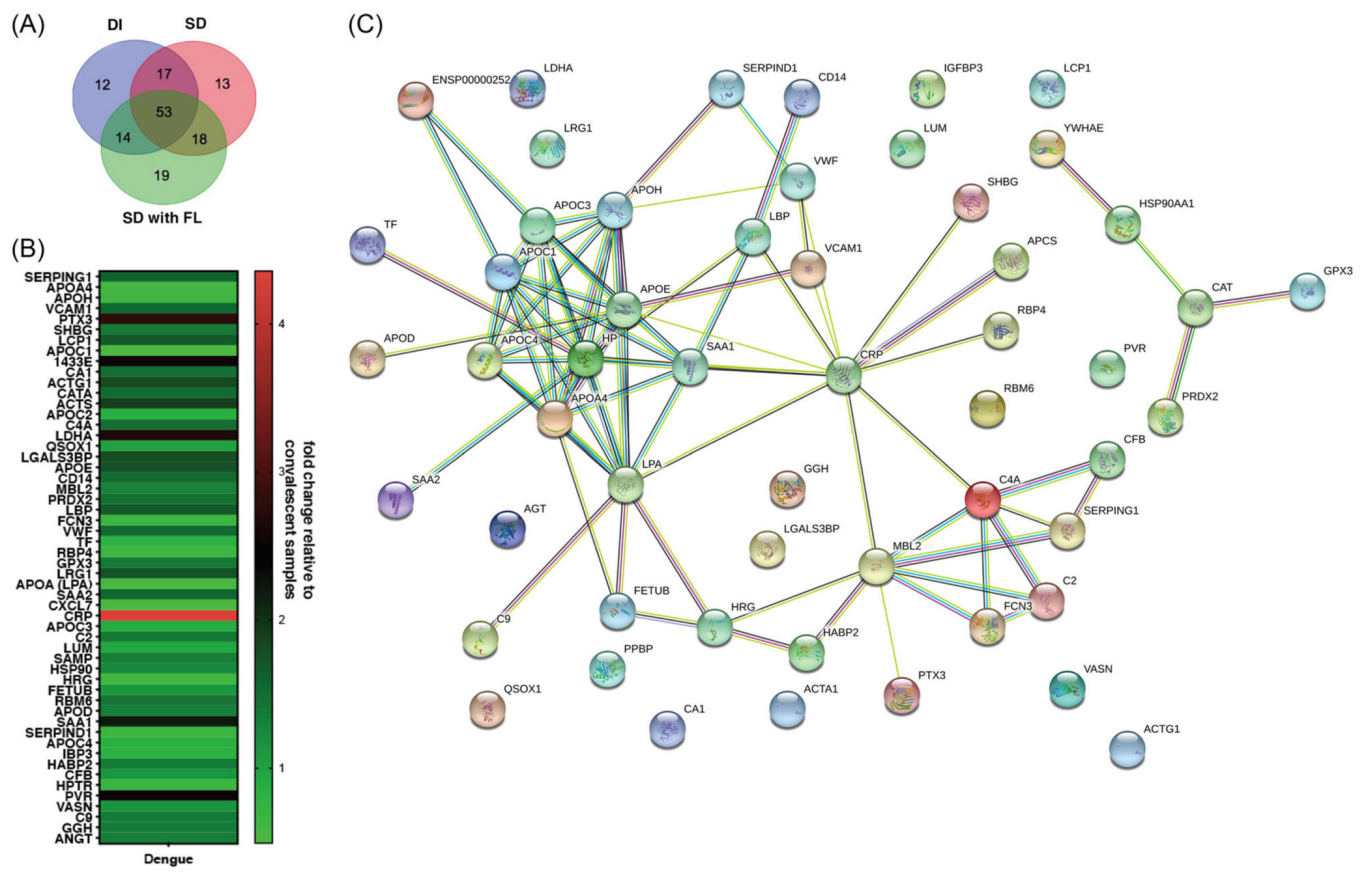
Protein identification and association network based on differential expressions in acute dengue with varying clinical symptoms. (A) Venn diagram showing the total number of proteins identified after combining all five sets of iTRAQ runs. (B) Heat map depicts 54 proteins that showed differential expression in acute dengue samples relative to convalescent samples (< 0.5- or > 1.5-fold) in at two out of the five iTRAQ runs. (C) STRING analysis with high confidence interaction score of 0.700 to identify the interacting nodes and pathways regulated by these 54 proteins.

**Figure 2:**
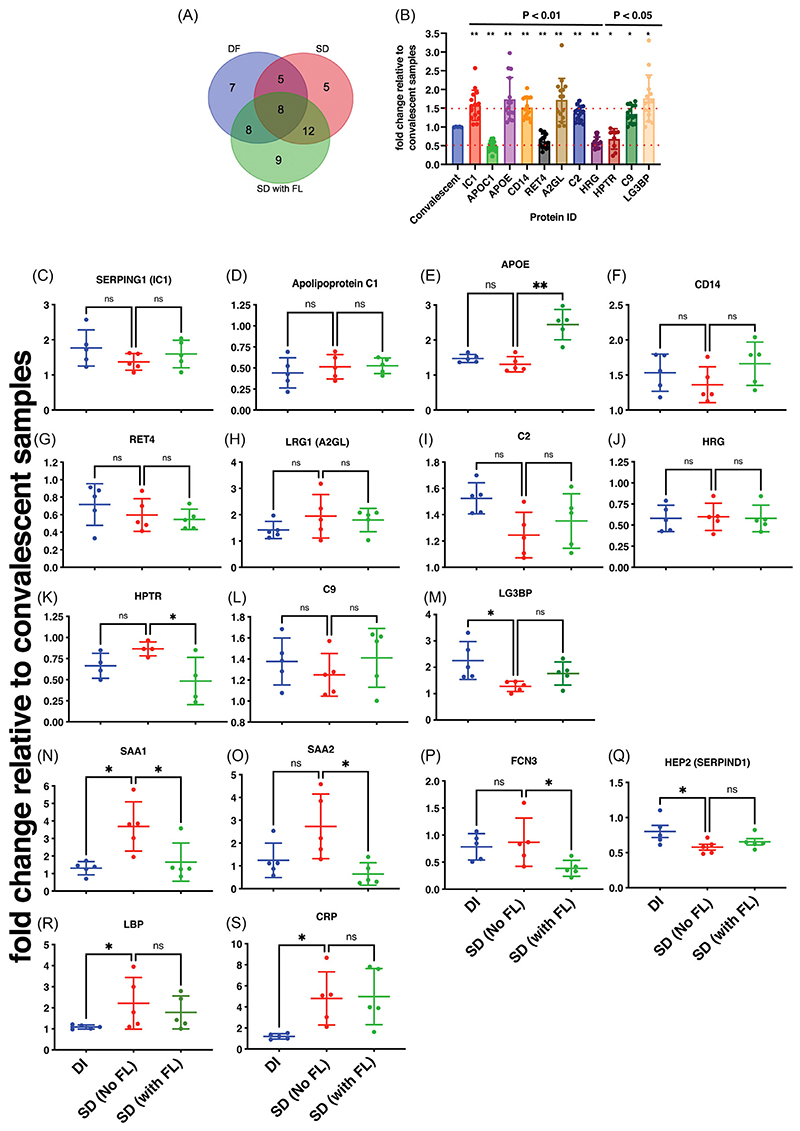
Proteins with differential expression identified in dengue plasma samples using iTRAQ approach. (A) Venn diagram showing the distribution of differentially regulated proteins among the dengue groups. (B) The graph indicates the proteins with significant modulation in dengue samples with respect to the convalescent group. (C–S) Graphs depict the proteins with significant modulation relative to SD (no fluid leak) condition. Statistical significance was determined by Kruskal-Wallis test with Dunn’s multiple comparison correction. *p < .05, **p < .01.

**Figure 3:**
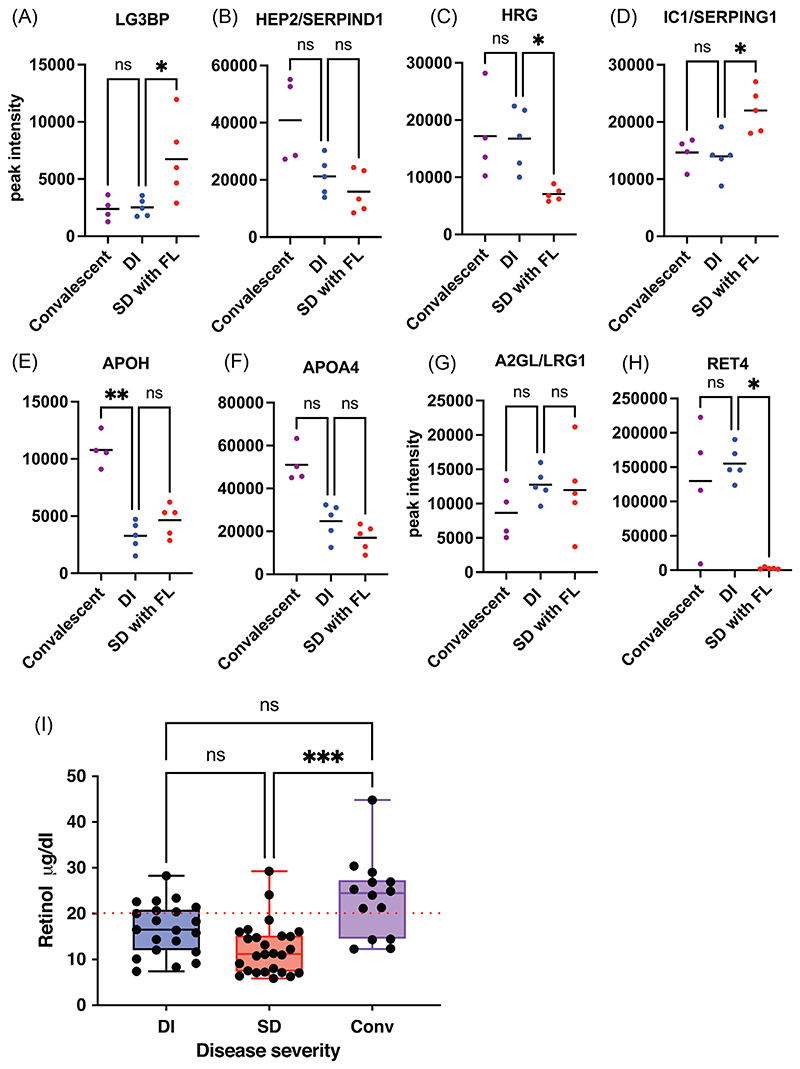
Validation of a subset of iTRAQ results using HR-MRM approach for the indicated proteins. (A-H) Peptides for each of the indicated proteins were used for HR-MRM to quantitate the peak intensity as a measure of abundance in each of the conditions, that is, convalescent (*n* = 4), mild dengue (DI) and severe dengue (*n* = 5 each). Mean values are indicated by a bar for each of the conditions. Abundance of peptides in convalescent or severe dengue was calculated relative to DI samples. (I) The concentration of retinol was determined in different samples from indicated dengue groups. Statistical significance was determined by Kruskal–Wallis test with Dunn’s multiple comparison correction using GraphPad prism software. *p < .05, **p < .01, ***p < .001, ns, nonsignificant.

**Figure 4:**
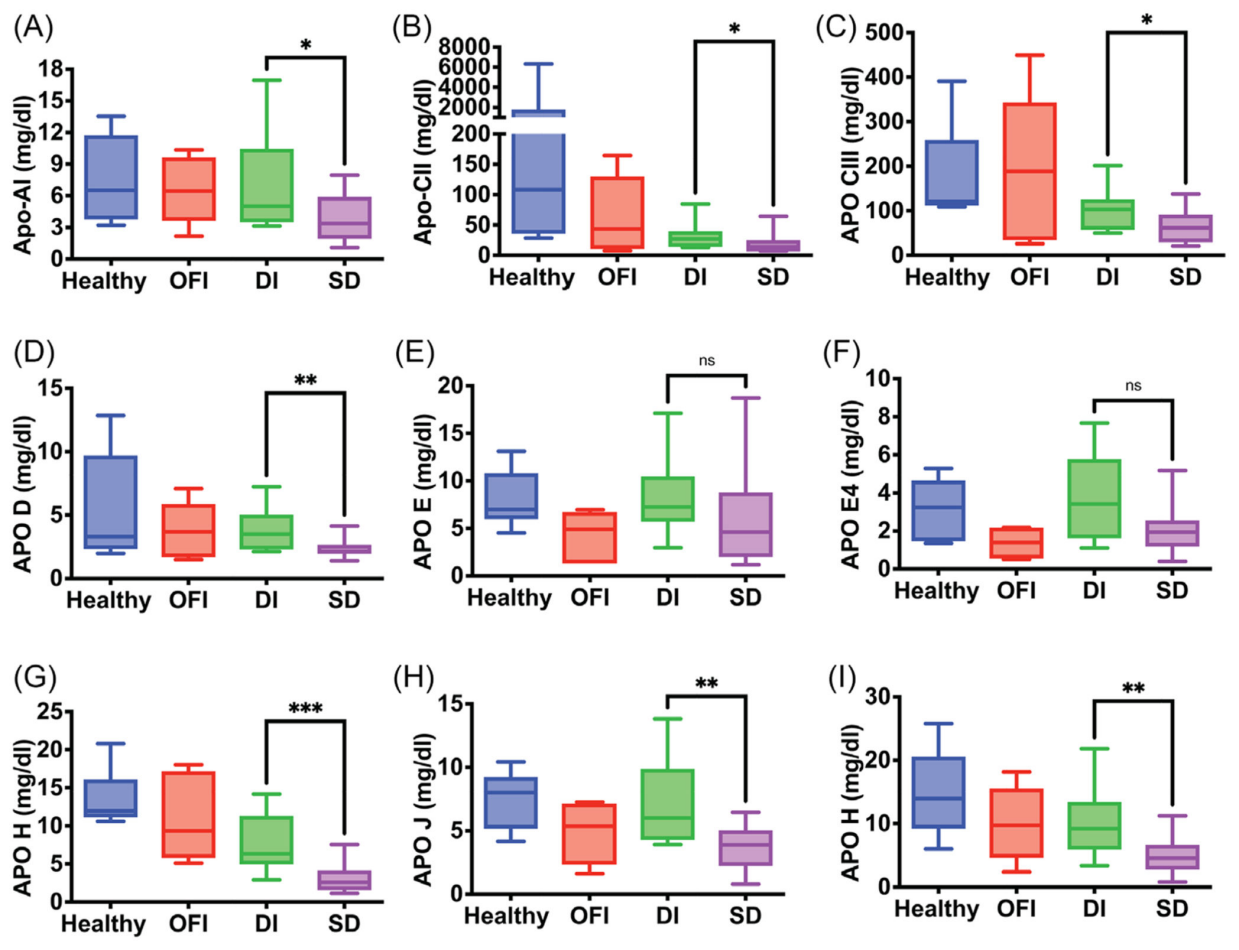
Quantitation and validation of human apolipoproteins in serum samples of dengue patients by multiplex bead assay. Different apolipoproteins (A–I) levels were determined in serum samples of mild dengue (DI, *n* = 12), severe dengue (SD, *n* = 15), and other febrile illness (OFI, *n* = 5), and healthy controls (*n* = 8) using multiplex bead-based assay panel. The concentrations of test samples were calculated by plotting the standard curve. Statistical significance was determined by Mann–Whitney test using GraphPad prism software. *p < .05, **p < .01, ***p < .001, ****p < .0001. Mild dengue (DI), severe dengue (SD), other febrile illness (OFI).
