# Supplementary material for: Severe dengue in children associates with dysregulation of lipid homeostasis, complement cascade and retinol transport
Source: Clin Transl Med. Author manuscript; Available in PMC 2023 Jun 7. (PMC10230155; doi:10.1002/ctm2.1271)
Supplement: Supplementary Discussion [file EMS176616-supplement-Supplementary_Discussion.docx]

**Supplementary Discussion**

In the present study, we utilized quantitative proteomic approaches for the identification and validation of proteins associating with severe dengue in children. The plasma lipid transport and complement pathways emerged as two prominent pathways from network analysis of 54 proteins which were dysregulated in dengue. Interestingly, we also found downregulation of retinol-binding protein which correlated with retinol levels in the plasma in dengue samples. Apolipoproteins are critical to maintain physiological lipid levels as they interact with receptors, enzymes and transporters involved in lipid metabolism ^1^. Apolipoproteins are the major protein constituents of high-density lipoproteins (HDLs) and a number of studies suggest modulation of HDL levels as an important and reliable marker for disease progression and outcomes in both communicable and non-communicable diseases (reviewed in ^2^). In the current study, using iTRAQ, MRM and quantitative ELISAs, we show that a subset of apolipoproteins, including the acute phase response apolipoproteins SAA1 and SAA2, are downregulated in severe dengue. Dysregulation of lipid homeostasis in dengue infection has been reported by previous studies. Lower levels of total cholesterol, high-density lipoproteins and low-density lipoproteins in plasma were associated with severe dengue infection ^3-5^. Our results are consistent with a previous report which has shown decrease in APOA1 levels in severe dengue by 2-D Fluorescence Difference Gel Electrophoresis ^6^. Interestingly, recent studies have identified a direct negative correlation between HDL-cholesterol and APOA1 levels and severe COVID-19 disease suggesting a broad antiviral or anti-inflammatory role for APOA1 and HDLs ^7^. APOA1 is the most widely studied member of the apolipoprotein family and has been reported to have antiviral activities for both enveloped and non-enveloped DNA and RNA viruses ^8,9^. APOA1 was shown to deplete lipid rafts and prevent DENV infection and the NS1 protein of DENV was reported to bind to APOA1 as a countermeasure ^10^. Recently, APOA1 mimetic peptides were shown to inhibit SARS-CoV-2 infection *in vitro* ^11^ and the same peptide was earlier shown to reduce the severity of influenza-mediated pneumonia in mice ^12^. APOA1 mimetic peptides were also shown to suppress interferon-induced inflammation and overexpression of APOA1 in mice played a protective effect against LPS-induced systemic inflammation and multiple organ damage ^13,14^. We speculate that intervention strategies to increase the APOA1 (and thereby HDL) levels in severe dengue patients may suppress inflammation and improve clinical outcomes. Further studies are needed to investigate the cause and effect of dyslipedemia in dengue infection.

The objective of the study was to identify markers that associate with severe dengue with or without hemorrhagic manifestations, we identified differential levels of RET4(RBP), HPTR, LG3BP, SAA1, SAA2, FCN3, HEP2, LBP, CRP, IC1, HRG and apolipoproteins in severe dengue relative to mild dengue. Two previous studies have identified LG3BP as upregulated protein in dengue samples however, no association was established with severe dengue ^15,16^. Our MRM data shows a significant upregulation of LG3BP in severe dengue samples. As LG3BP plays an important role in innate immune responses and cell-cell adhesion, its role in dengue pathogenesis needs to be characterized further. Histidine-rich glycoprotein, a multi-functional plasma glycoprotein, was downregulated in all the three conditions of dengue in iTRAQ assays. Validation by HR-MRM showed a specific downregulation in severe dengue which is consistent with previous reports which showed downregulation of HRG in severe dengue by two-dimensional difference gel electrophoresis ^6^. HRG levels were also found to be downregulated in plasma samples of rhesus macaques infected with simian immunodeficiency virus during acute phase but not during the chronic phase of infection^17^. Interestingly, HRG was shown to exhibit a broad range of direct antiviral activity by interacting with HIV particles and was also able to inhibit RSV and HSV-2 under acidic conditions ^18^. More recently, HRG was found to be more abundant in the serum samples of COVID-19 survivors as compared to non-survivors ^19^. HRG functions as an adaptor molecule and through its multidomain interactions with variety of ligands which include heparin, phospholipids, immune complexes, complement proteins and metal ions, HRG regulates diverse physiological functions critical to immunity such as clearance of immune complexes, apoptotic cells, coagulation and has also emerged as a key player in vascular biology ^20,21^. Therefore, further studies on the role of HRG in dengue infection may shed light on the pathogenesis of severe dengue.

Plasma protease C1 inhibitor (IC1) regulates complement cascade by inhibiting C1r and C1s, the first components of complement cascade. We found upregulation of IC1 in all the three conditions of dengue in iTRAQ. However, HR-MRM showed significant upregulation only in SD samples. Ficolins regulate the lectin pathway of complement system and have been shown to inhibit some of the RNA viruses ^22^. We identified downregulation of FCN3 in severe dengue with fluid leak samples. A recent study from Vietnam had reported upregulation of ficolin-2 (FCN2) during acute phase of dengue, however, dengue with warning signs samples had lower levels of FCN2 as compared to dengue fever patients ^23^. Complement proteins have been shown to physically interact with dengue virion and liver tissues from autopsy of dengue patients also showed deposition of complement cascade proteins ^24-26^. Dengue virus non-structural protein 1 was shown to activate complement cascade and NS1 levels in samples from dengue shock syndrome associated with components of complement activation cascade ^27^ suggesting a link between severe dengue and complement pathway. However, complement activation is also an important component of host response to viral infections and the beneficial effect of complement pathway in resolving flavivirus infections has also been demonstrated by multiple studies (reviewed in ^28^). Therefore, the NS1 protein of DENV and other flaviviruses is also implicated in evading the complement pathway by binding to multiple components of complement cascades such as Factor H ^29^, C1q ^30^, C4b binding protein ^31^, mannose-binding lectin ^32^ to prevent the activation of complement cascade. Interestingly, HDLs were found to be associated with complement proteins and were proposed to regulate inflammation via complement cascade ^33^. Therefore, the dynamics of complement activation and its regulation during acute phase of dengue infection may be a critical determinant of clinical outcomes.

The levels of retinol-binding protein, RET4 (or RBP/RBP4) was downregulated across dengue disease severity in iTRAQ experiments. However, HR-MRM data showed a significant downregulation of the RET4 peptide in severe dengue samples relative to mild dengue or convalescent samples. Retinol levels go down in the circulation as part of the acute phase response during infection and inflammation ^34-36^. Lower levels of RBP and Vitamin D are associated with severe outcomes in children hospitalized for lower respiratory tract infection with respiratory syncytial virus and human metapneumovirus ^37^. Recent reports have shown association of lower levels of RBP4 with critically-ill, hospitalized COVID-19 patients and retinol levels were also low in acute phase of COVID-19 ^38^. RBP levels were found to be lower in clinically apparent dengue patients as compared to non-febrile dengue cases ^39^. A previous study from Guatemala has shown lower levels of plasma retinol in dengue patients compared to the controls ^40^. The importance of micronutrients in influencing infectious disease outcomes has been proven by a number of supplementation trials. The risks of mortality and morbidity was found to be reduced upon Vitamin A supplementation in a number of diseases such as measles, diarrhea and HIV which is supported by the findings that diverse immunological functions are regulated by Vitamin A including maturation of dendritic cells, cell-mediated immunity, maintaining the integrity of epithelial barrier, T-cell proliferation ^41-43^. Retinoic acid, a vitamin A metabolite, has been shown be essential for CD4^+^ effector T cell functions ^44^. Lower levels of Vitamin A in dengue may affect the innate and antibody responses. Therefore, measuring the impact of Vitamin A deficiency on dengue disease outcomes and assessing the effect of vitamin A supplementation in dengue patients would be a prudent step for augmenting the clinical management in a cost-effective manner.

Liver involvement in dengue worsens the prognosis to recovery in dengue infection ^45^. Dengue causes liver damage by directly affecting hepatocyte and Kupffer cell functions, by cytokine storm, and causing decreased hepatic perfusion ^46,47^. We have markers of acute phase response, observed dyslipidaemia, aberrant complement activation and lower levels of RBP and retinol which are all functions regulated by liver. Our study provides insights into some of the liver functions that could be targeted for improving the clinical outcomes of severe dengue.

**References for Supplementary discussion**

1. Ramasamy I. Recent advances in physiological lipoprotein metabolism. *Clinical Chemistry and Laboratory Medicine (CCLM)* 2014; **52**(12): 1695-727.

2. Cho KH. The Current Status of Research on High-Density Lipoproteins (HDL): A Paradigm Shift from HDL Quantity to HDL Quality and HDL Functionality. *Int J Mol Sci* 2022; **23**(7).

3. Biswas HH, Gordon A, Nunez A, Perez MA, Balmaseda A, Harris E. Lower Low-Density Lipoprotein Cholesterol Levels Are Associated with Severe Dengue Outcome. *PLoS Negl Trop Dis* 2015; **9**(9): e0003904.

4. van Gorp EC, Suharti C, Mairuhu AT, et al. Changes in the plasma lipid profile as a potential predictor of clinical outcome in dengue hemorrhagic fever. *Clin Infect Dis* 2002; **34**(8): 1150-3.

5. Marin-Palma D, Sirois CM, Urcuqui-Inchima S, Hernandez JC. Inflammatory status and severity of disease in dengue patients are associated with lipoprotein alterations. *PLoS One* 2019; **14**(3): e0214245.

6. Albuquerque LM, Trugilho MR, Chapeaurouge A, et al. Two-dimensional difference gel electrophoresis (DiGE) analysis of plasmas from dengue fever patients. *J Proteome Res* 2009; **8**(12): 5431-41.

7. Sun JT, Chen Z, Nie P, et al. Lipid Profile Features and Their Associations With Disease Severity and Mortality in Patients With COVID-19. *Front Cardiovasc Med* 2020; **7**: 584987.

8. Singh IP, Chopra AK, Coppenhaver DH, Ananatharamaiah GM, Baron S. Lipoproteins account for part of the broad non-specific antiviral activity of human serum. *Antiviral Res* 1999; **42**(3): 211-8.

9. Srinivas RV, Birkedal B, Owens RJ, Anantharamaiah GM, Segrest JP, Compans RW. Antiviral effects of apolipoprotein A-I and its synthetic amphipathic peptide analogs. *Virology* 1990; **176**(1): 48-57.

10. Coelho DR, Carneiro PH, Mendes-Monteiro L, et al. ApoA1 Neutralizes Proinflammatory Effects of Dengue Virus NS1 Protein and Modulates Viral Immune Evasion. *J Virol* 2021; **95**(13): e0197420.

11. Kelesidis T, Madhav S, Petcherski A, et al. The ApoA-I mimetic peptide 4F attenuates in vitro replication of SARS-CoV-2, associated apoptosis, oxidative stress and inflammation in epithelial cells. *Virulence* 2021; **12**(1): 2214-27.

12. Van Lenten BJ, Wagner AC, Navab M, et al. D-4F, an apolipoprotein A-I mimetic peptide, inhibits the inflammatory response induced by influenza A infection of human type II pneumocytes. *Circulation* 2004; **110**(20): 3252-8.

13. Fernandez-Sendin M, Di Trani CA, Bella A, et al. Long-Term Liver Expression of an Apolipoprotein A-I Mimetic Peptide Attenuates Interferon-Alpha-Induced Inflammation and Promotes Antiviral Activity. *Front Immunol* 2020; **11**: 620283.

14. Li Y, Dong JB, Wu MP. Human ApoA-I overexpression diminishes LPS-induced systemic inflammation and multiple organ damage in mice. *Eur J Pharmacol* 2008; **590**(1-3): 417-22.

15. Liu KT, Liu YH, Chen YH, et al. Serum Galectin-9 and Galectin-3-Binding Protein in Acute Dengue Virus Infection. *Int J Mol Sci* 2016; **17**(6).

16. Fragnoud R, Yugueros-Marcos J, Pachot A, Bedin F. Isotope Coded Protein Labeling analysis of plasma specimens from acute severe dengue fever patients. *Proteome science* 2012; **10**(1): 60-.

17. Wiederin JL, Donahoe RM, Anderson JR, et al. Plasma proteomic analysis of simian immunodeficiency virus infection of rhesus macaques. *J Proteome Res* 2010; **9**(9): 4721-31.

18. Dantas E, Erra Diaz F, Pereyra Gerber P, et al. Histidine-Rich Glycoprotein Inhibits HIV-1 Infection in a pH-Dependent Manner. *J Virol* 2019; **93**(4).

19. Vollmy F, van den Toorn H, Zenezini Chiozzi R, et al. A serum proteome signature to predict mortality in severe COVID-19 patients. *Life Sci Alliance* 2021; **4**(9).

20. Poon IK, Patel KK, Davis DS, Parish CR, Hulett MD. Histidine-rich glycoprotein: the Swiss Army knife of mammalian plasma. *Blood* 2011; **117**(7): 2093-101.

21. Wakabayashi S. Chapter Nine - New Insights into the Functions of Histidine-Rich Glycoprotein. In: Jeon KW, ed. International Review of Cell and Molecular Biology: Academic Press; 2013: 467-93.

22. Mason CP, Tarr AW. Human lectins and their roles in viral infections. *Molecules* 2015; **20**(2): 2229-71.

23. Giang NT, Tong HV, Nghia TH, et al. Association of FCN2 polymorphisms and Ficolin-2 levels with dengue fever in Vietnamese patients. *Int J Infect Dis* 2020; **95**: 253-61.

24. Fragnoud R, Flamand M, Reynier F, et al. Differential proteomic analysis of virus-enriched fractions obtained from plasma pools of patients with dengue fever or severe dengue. *BMC Infect Dis* 2015; **15**: 518.

25. Khadka S, Vangeloff AD, Zhang C, et al. A physical interaction network of dengue virus and human proteins. *Mol Cell Proteomics* 2011; **10**(12): M111 012187.

26. Aye KS, Charngkaew K, Win N, et al. Pathologic highlights of dengue hemorrhagic fever in 13 autopsy cases from Myanmar. *Human Pathology* 2014; **45**(6): 1221-33.

27. Avirutnan P, Punyadee N, Noisakran S, et al. Vascular leakage in severe dengue virus infections: a potential role for the nonstructural viral protein NS1 and complement. *J Infect Dis* 2006; **193**(8): 1078-88.

28. Conde JN, Silva EM, Barbosa AS, Mohana-Borges R. The Complement System in Flavivirus Infections. *Frontiers in Microbiology* 2017; **8**.

29. Chung KM, Liszewski MK, Nybakken G, et al. West Nile virus nonstructural protein NS1 inhibits complement activation by binding the regulatory protein factor H. *Proc Natl Acad Sci U S A* 2006; **103**(50): 19111-6.

30. Silva EM, Conde JN, Allonso D, Nogueira ML, Mohana-Borges R. Mapping the interactions of dengue virus NS1 protein with human liver proteins using a yeast two-hybrid system: identification of C1q as an interacting partner. *PLoS One* 2013; **8**(3): e57514.

31. Avirutnan P, Hauhart RE, Somnuke P, Blom AM, Diamond MS, Atkinson JP. Binding of flavivirus nonstructural protein NS1 to C4b binding protein modulates complement activation. *J Immunol* 2011; **187**(1): 424-33.

32. Thiemmeca S, Tamdet C, Punyadee N, et al. Secreted NS1 Protects Dengue Virus from Mannose-Binding Lectin-Mediated Neutralization. *J Immunol* 2016; **197**(10): 4053-65.

33. Vaisar T, Pennathur S, Green PS, et al. Shotgun proteomics implicates protease inhibition and complement activation in the antiinflammatory properties of HDL. *J Clin Invest* 2007; **117**(3): 746-56.

34. Bresnahan KA, Tanumihardjo SA. Undernutrition, the acute phase response to infection, and its effects on micronutrient status indicators. *Adv Nutr* 2014; **5**(6): 702-11.

35. Schweigert FJ. Inflammation-induced changes in the nutritional biomarkers serum retinol and carotenoids. *Current Opinion in Clinical Nutrition & Metabolic Care* 2001; **4**(6).

36. Rosales FJ, Ritter SJ, Zolfaghari R, Smith JE, Ross AC. Effects of acute inflammation on plasma retinol, retinol-binding protein, and its mRNA in the liver and kidneys of vitamin A-sufficient rats. *Journal of Lipid Research* 1996; **37**(5): 962-71.

37. Hurwitz JL, Jones BG, Penkert RR, et al. Low Retinol-Binding Protein and Vitamin D Levels Are Associated with Severe Outcomes in Children Hospitalized with Lower Respiratory Tract Infection and Respiratory Syncytial Virus or Human Metapneumovirus Detection. *J Pediatr* 2017; **187**: 323-7.

38. Vollenberg R, Tepasse PR, Fobker M, Husing-Kabar A. Significantly Reduced Retinol Binding Protein 4 (RBP4) Levels in Critically Ill COVID-19 Patients. *Nutrients* 2022; **14**(10).

39. Finkelstein JL, Colt S, Layden AJ, et al. Micronutrients, Immunological Parameters, and Dengue Virus Infection in Coastal Ecuador: A Nested Case-Control Study in an Infectious Disease Surveillance Program. *J Infect Dis* 2020; **221**(1): 91-101.

40. Klassen P, Biesalski HK, Mazariegos M, Solomons NW, Fürst P. Classic dengue fever affects levels of circulating antioxidants. *Nutrition* 2004; **20**(6): 542-7.

41. Beijer MR, Kraal G, den Haan JM. Vitamin A and dendritic cell differentiation. *Immunology* 2014; **142**(1): 39-45.

42. Ross AC, Stephensen CB. Vitamin A and retinoids in antiviral responses. *FASEB J* 1996; **10**(9): 979-85.

43. Villamor E, Fawzi WW. Effects of vitamin a supplementation on immune responses and correlation with clinical outcomes. *Clin Microbiol Rev* 2005; **18**(3): 446-64.

44. Hall JA, Cannons JL, Grainger JR, et al. Essential role for retinoic acid in the promotion of CD4(+) T cell effector responses via retinoic acid receptor alpha. *Immunity* 2011; **34**(3): 435-47.

45. Parkash O, Almas A, Jafri SMW, Hamid S, Akhtar J, Alishah H. Severity of acute hepatitis and its outcome in patients with dengue fever in a tertiary care hospital Karachi, Pakistan (South Asia). *BMC Gastroenterology* 2010; **10**(1): 43.

46. Seneviratne SL, Malavige GN, de Silva HJ. Pathogenesis of liver involvement during dengue viral infections. *Trans R Soc Trop Med Hyg* 2006; **100**(7): 608-14.

47. Samanta J, Sharma V. Dengue and its effects on liver. *World J Clin Cases* 2015; **3**(2): 125-31.
