# Supplementary material for: Severe dengue in children associates with dysregulation of lipid homeostasis, complement cascade and retinol transport
Source: Clin Transl Med. Author manuscript; Available in PMC 2023 Jun 7. (PMC10230155; doi:10.1002/ctm2.1271)
Supplement: Supplementary Figure Legends [file EMS176616-supplement-Supplementary_Figure_Legends.docx]

**Figure legends for supplementary data**

**Supplementary Figure S1: Workflow for quantitative proteomics using iTRAQ reagent.** Equal concentration of plasma protein was used for convalescent, dengue fever, SD with and without fluid leakage conditions. Abundant proteins in the plasma samples were depleted using a commercial depletion column kit. Samples were labeled with iTRAQ reporter labels, pooled, and subjected to strong cation exchange chromatography. LC-MS/MS was performed for protein identification and quantification. Abbreviations: severe dengue without fluid leakage (SD w/o FL), severe dengue with fluid leakage (SD with FL).

**Supplementary Figure S2: Peptide counts of differentially regulated proteins in dengue infection**. Pie chart showing the distribution of peptide counts across proteins that are differentially regulated in dengue infection categorized as per disease symptoms.

**Supplementary Figure S3: Pathway network of differentially regulated proteins**. Reactome database-based connection of different but related pathways that were enriched in dengue-infected samples using network plots.

**Supplementary Figure S4: Activation of the complement cascade in dengue infection**. Pathway analysis of differentially expressed proteins indicates the networks leading to the activation of the complement cascade.

**Supplementary Figure S5: Identification of plasma lipoprotein assembly network in dengue infection**. Pathway analysis of differentially regulated proteins identifies the proteins involved in plasma lipoprotein assembly, remodeling, and clearance.

**Supplementary Figure S6: Clinical parameters of samples used in MRM:** 10 samples from convalescent (CONV), mild dengue and severe dengue patients were used in validation of iTRAQ data. (A) Levels of NS-1 antigen as measured by ELISA (Panbio Units) is shown. (B) Platelet counts in mild and severe dengue samples. ND: Not determined. (C) DENV RNA levels in the samples were measured by qRT-PCR. (D) Day of fever (DOF) at the time of enrolment is shown. P values were estimated by two-tailed, non-parametric, Mann-Whitney test. ** P<0.01, ***P<0.001.

**Supplementary Figure S7: Validation of Galectin-3-binding protein (LG3BP) using HR-MRM approach**. The figure shows the co-elution profile of all the transitions for a peptide (VEIFYR) in protein LG3BP. All transitions for this specific peptide coelute confirm peptide identification. The graph indicates the expression of LG3BP among the dengue groups.

**Supplementary Figure S8: Validation of Apolipoprotein H using HR-MRM approach**. The figure shows signal intensities of extracted ion current ( XIC) of the peptide FICPLTGLWPINTLK with protein APOH in the upper panel. Lower left panel shows quite stable retention times and the horizontal peak apex lines match up for all transitions in all the replicates. All transitions for this specific peptide coelute confirming peptide identification. The graph indicates the expression of APOH among the different dengue groups. The bars shows the mean average value among the replicates and whisker represent one standard deviation to either side of the mean.

**Supplementary Figure S9: Validation of Apolipoprotein A-IV using HR-MRM approach**. Upper panel shows XIC of the targeted peptide SLAELGGHLDQQVEEFR from Apolipoprotein A-IV protein generated post-acquisition. Lower right panel shows the relative quantitation of the peptide with dot product (dotp) values. The dotp values shows the similarity between the library spectrum peaks and the measured peak areas. The contribution from each distinct fragment ion is shown as a different color in the bars. Real quantitative data has been obtained, as there is no interfering signal inside the integration boundaries. There is strong, clean and co-eluting peaks for all the transitions. Lower left panel shows the peak areas among the groups. The bars shows the mean average value among the replicates and whisker represent one standard deviation to either side of the mean.

**Supplementary Figure S10: Validation of LRG-1 using HR-MRM approach**. The figure shows the co-elution profile of all the transitions for a peptide (VAAGAFQGLR) from protein LRG-1. All transitions for this specific peptide coelute confirm peptide identification. Lower left panel shows that the relative ion abundances are stable in the peak areas views. The graph indicates the expression of LRG-1 among the dengue groups.

**Supplementary Figure S11: Validation of LRG-1 using ELISA.** LRG-1 was determined in serum samples of patients with healthy control (n=8), mild dengue (DI, n=12), severe dengue (SD, n=15), and other febrile illness (OFI, n=6). The analysis was carried out by quantitative ELISA (Raybiotech) as per the manufacturer’s instructions. Error bars indicate mean with SD. Statistical significance was determined by non-parametric Kruskal-Wallis using GraphPad prism software. *p=0.0135; ns-non-significant.

**Supplementary Figure S12: Downregulation of Retinol-binding protein 4 (RET4) in severe dengue samples**. The figure from HR-MRM run shows the co-elution profile of all the transitions for a peptide (YWGVASFLQK) in protein RET4. All transitions for this specific peptide coelute confirm peptide identification. The graph indicates the expression of RET4 among the dengue groups.
