# Supplementary figures and images for: Severe dengue in children associates with dysregulation of lipid homeostasis, complement cascade and retinol transport
Source: Clin Transl Med. Author manuscript; Available in PMC 2023 Jun 7. (PMC10230155; doi:10.1002/ctm2.1271)

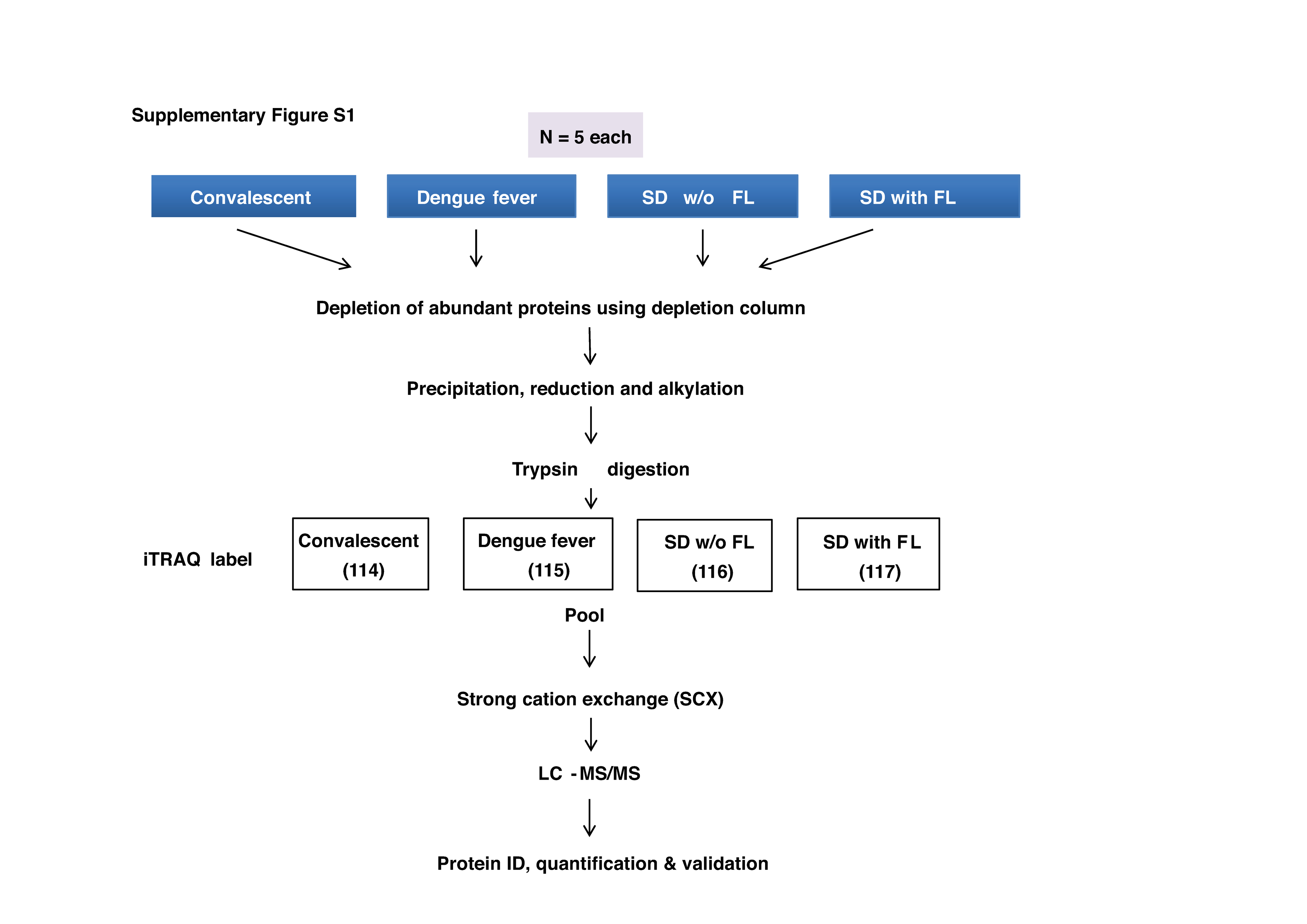

Supplement: Supplementary Figure S1 [file EMS176616-supplement-Supplementary_Figure_S1.tif]

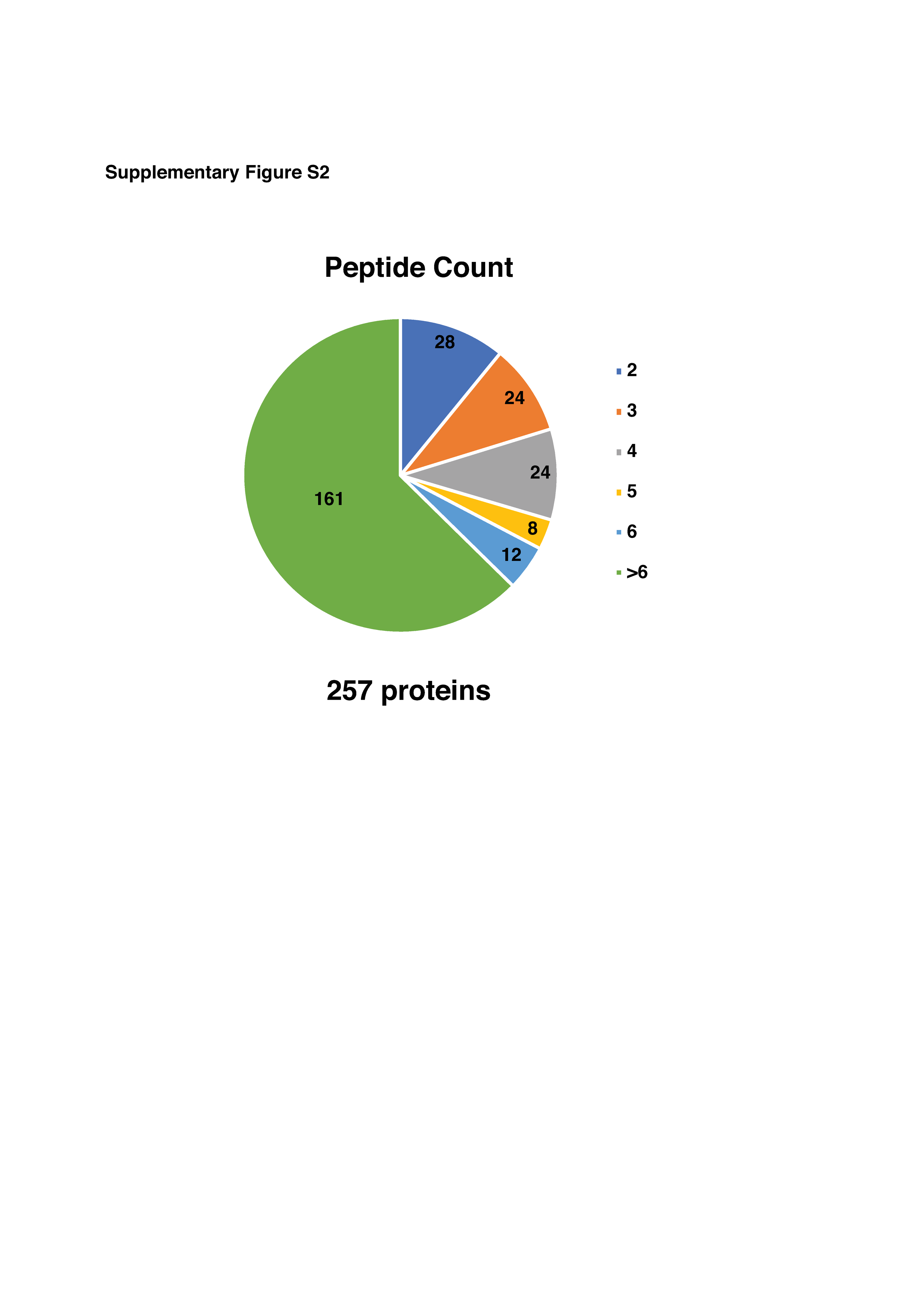

Supplement: Supplementary Figure S2 [file EMS176616-supplement-Supplementary_Figure_S2.tif]

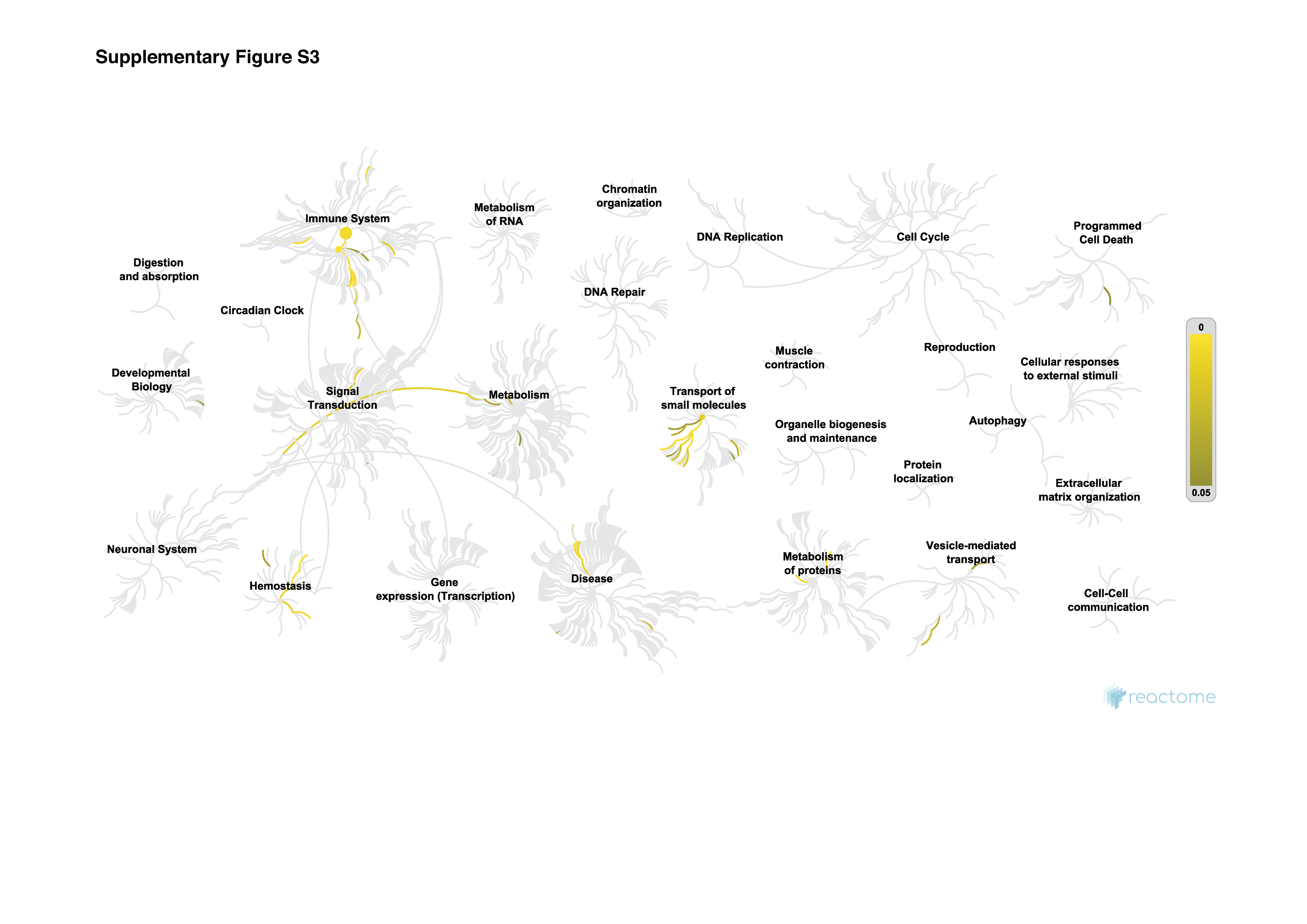

Supplement: Supplementary Figure S3 [file EMS176616-supplement-Supplementary_Figure_S3.tif]

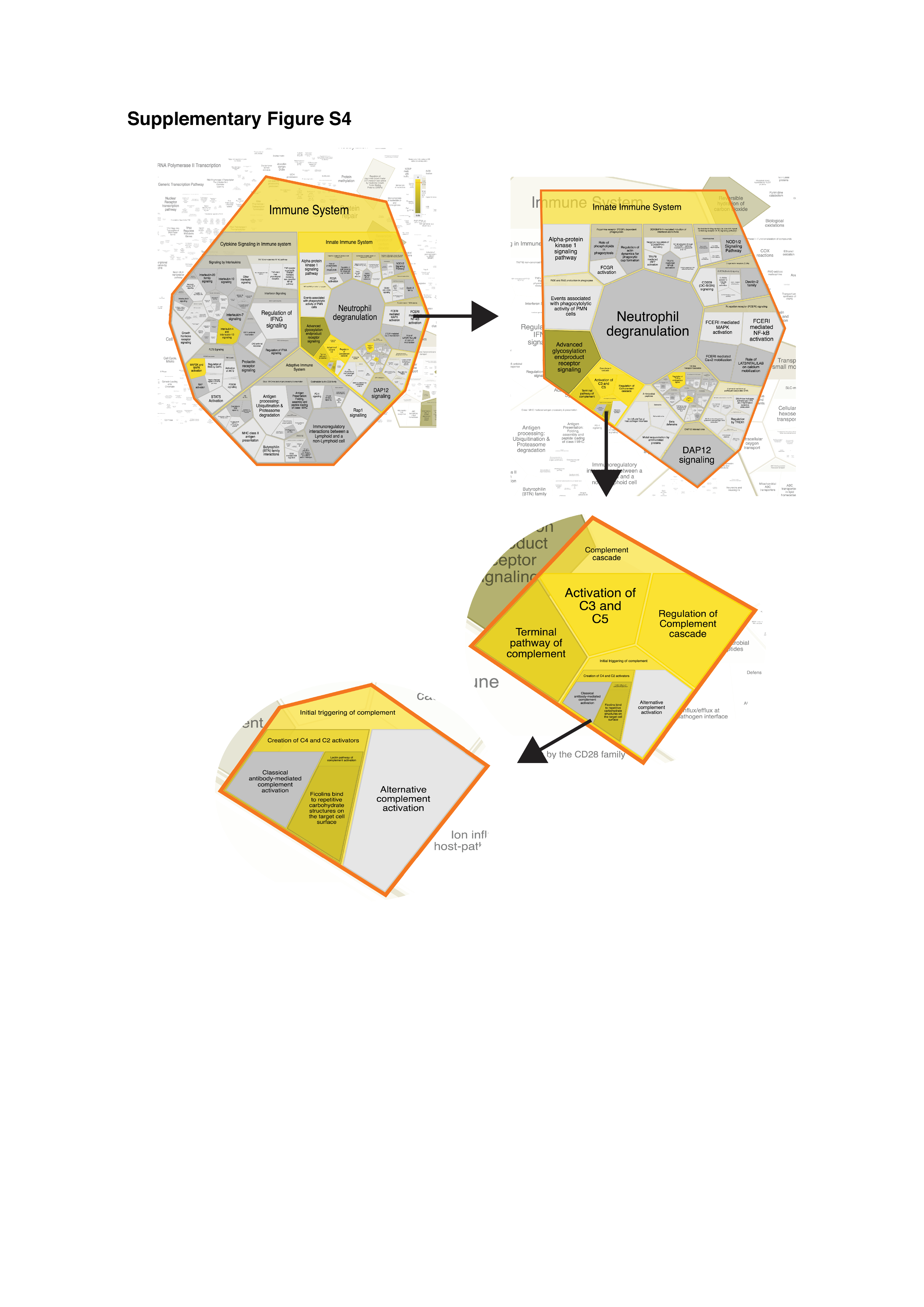

Supplement: Supplementary Figure S4 [file EMS176616-supplement-Supplementary_Figure_S4.tif]

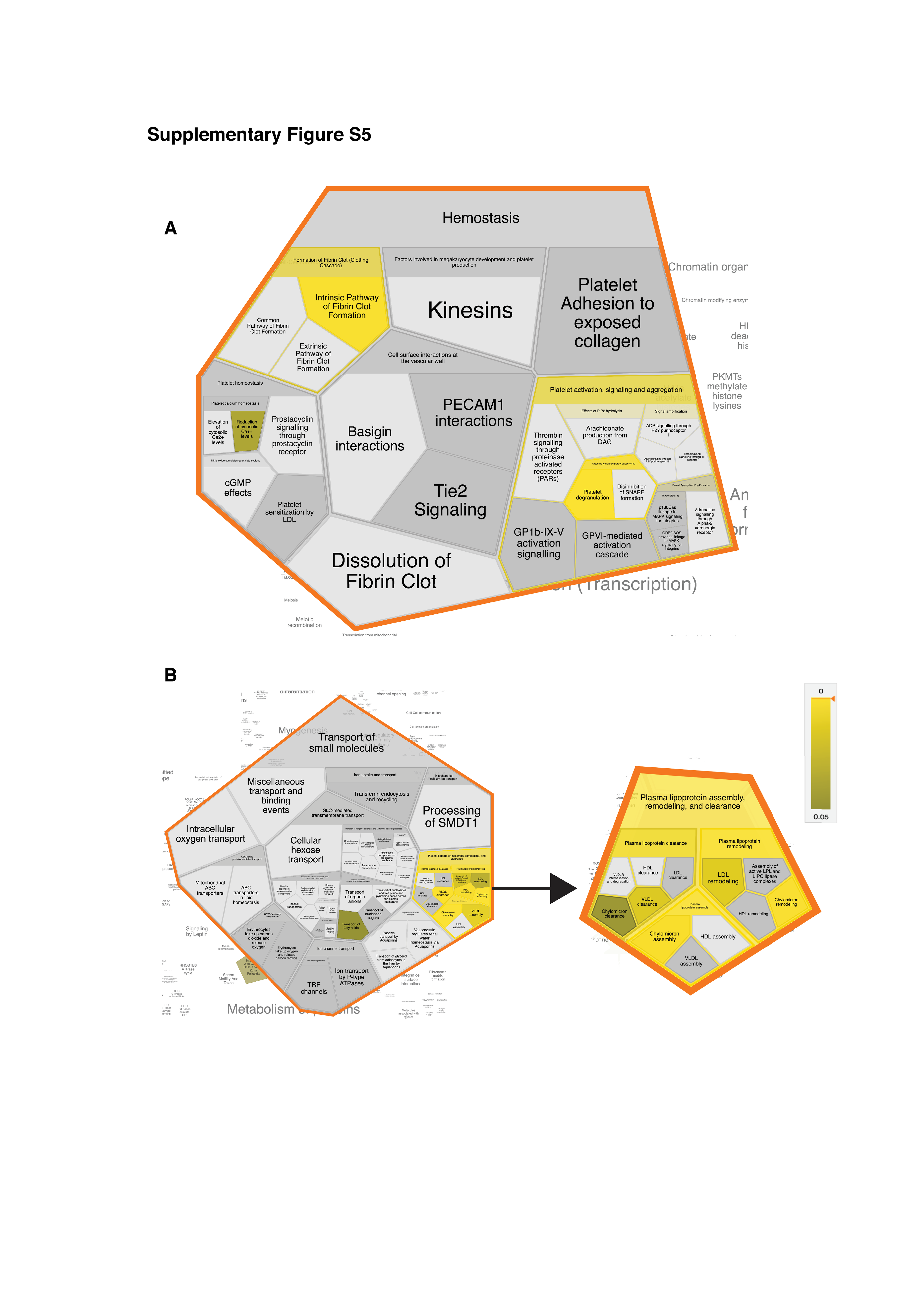

Supplement: Supplementary Figure S5 [file EMS176616-supplement-Supplementary_Figure_S5.tif]

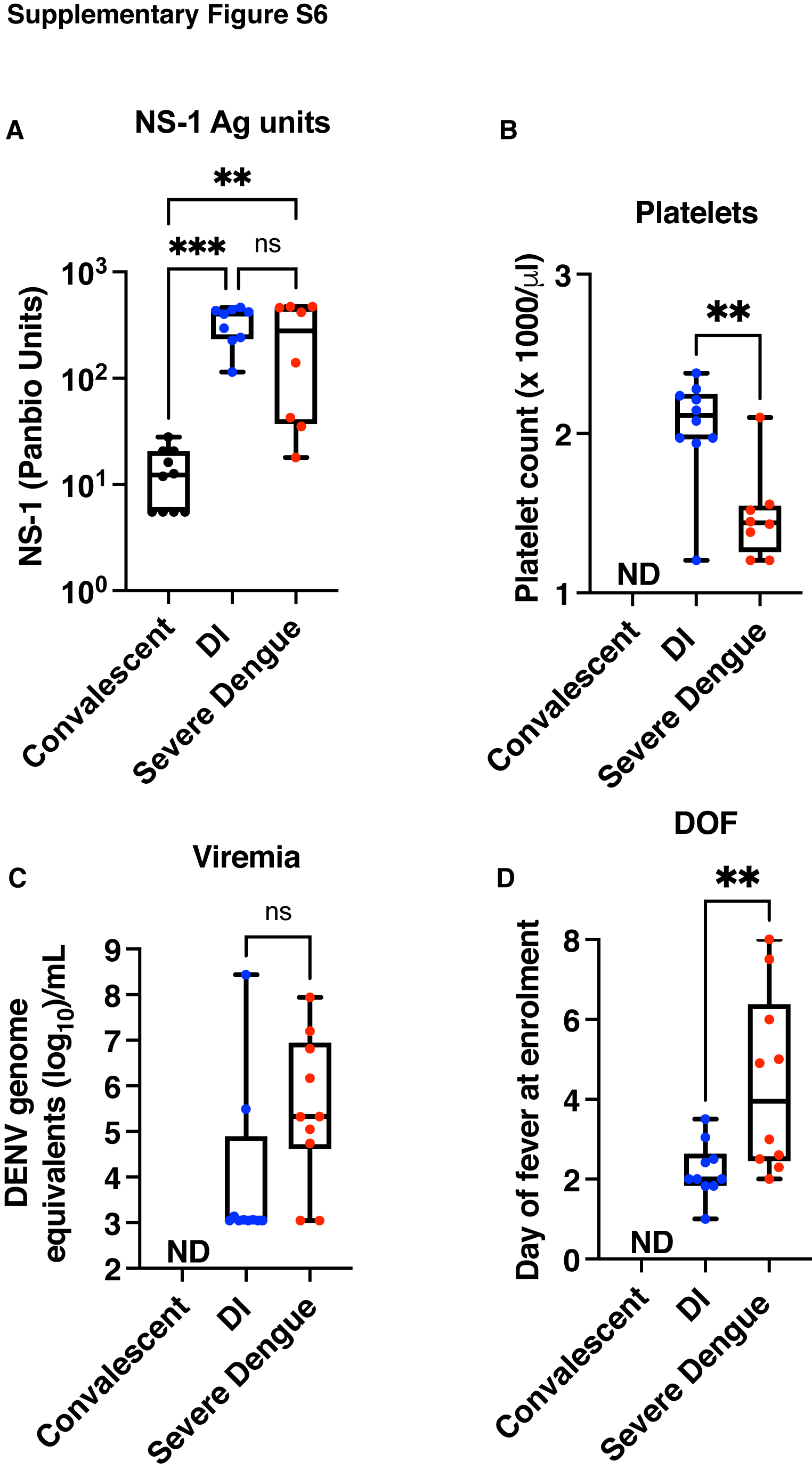

Supplement: Supplementary Figure S6 [file EMS176616-supplement-Supplementary_Figure_S6.tif]

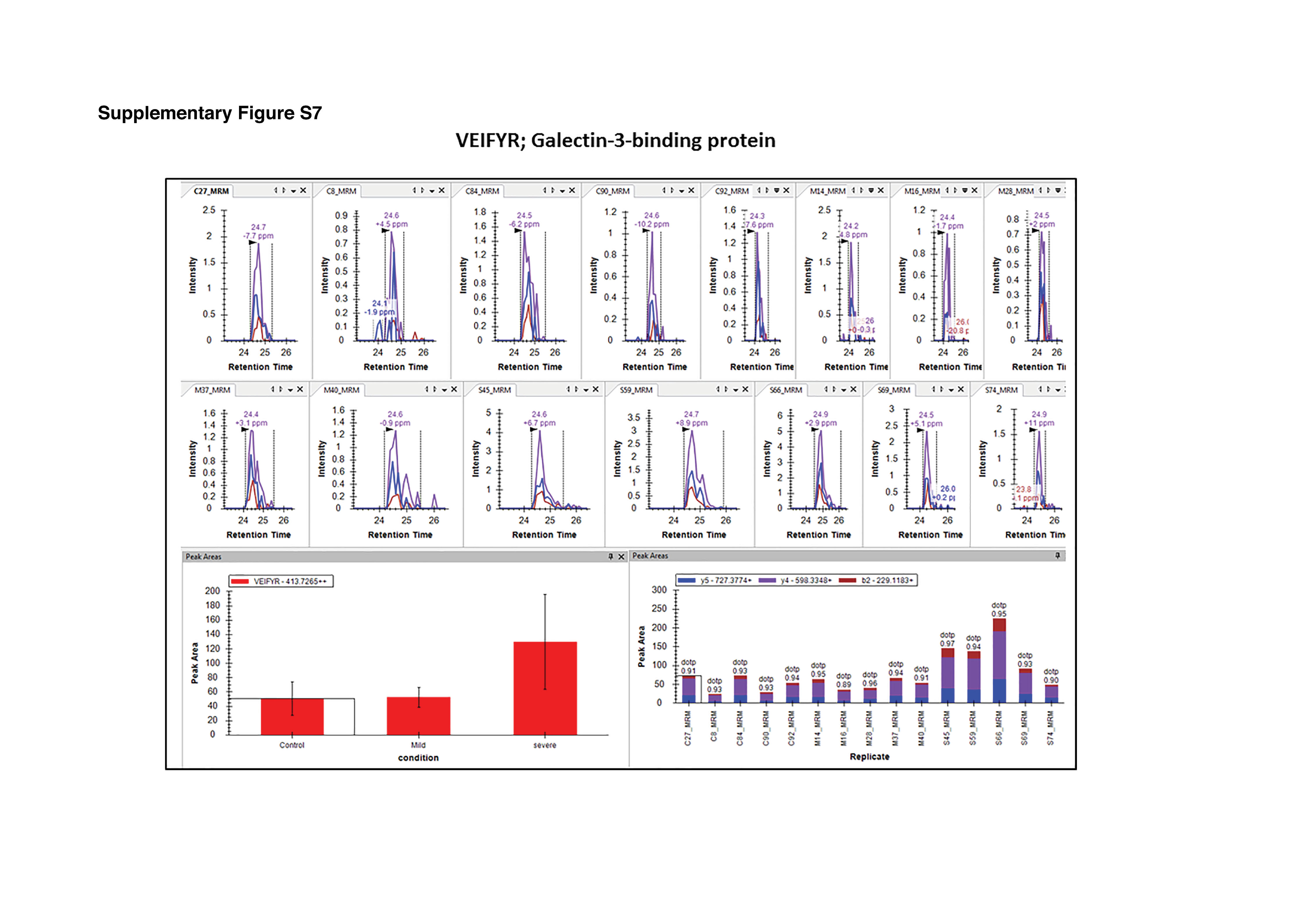

Supplement: Supplementary Figure S7 [file EMS176616-supplement-Supplementary_Figure_S7.tif]

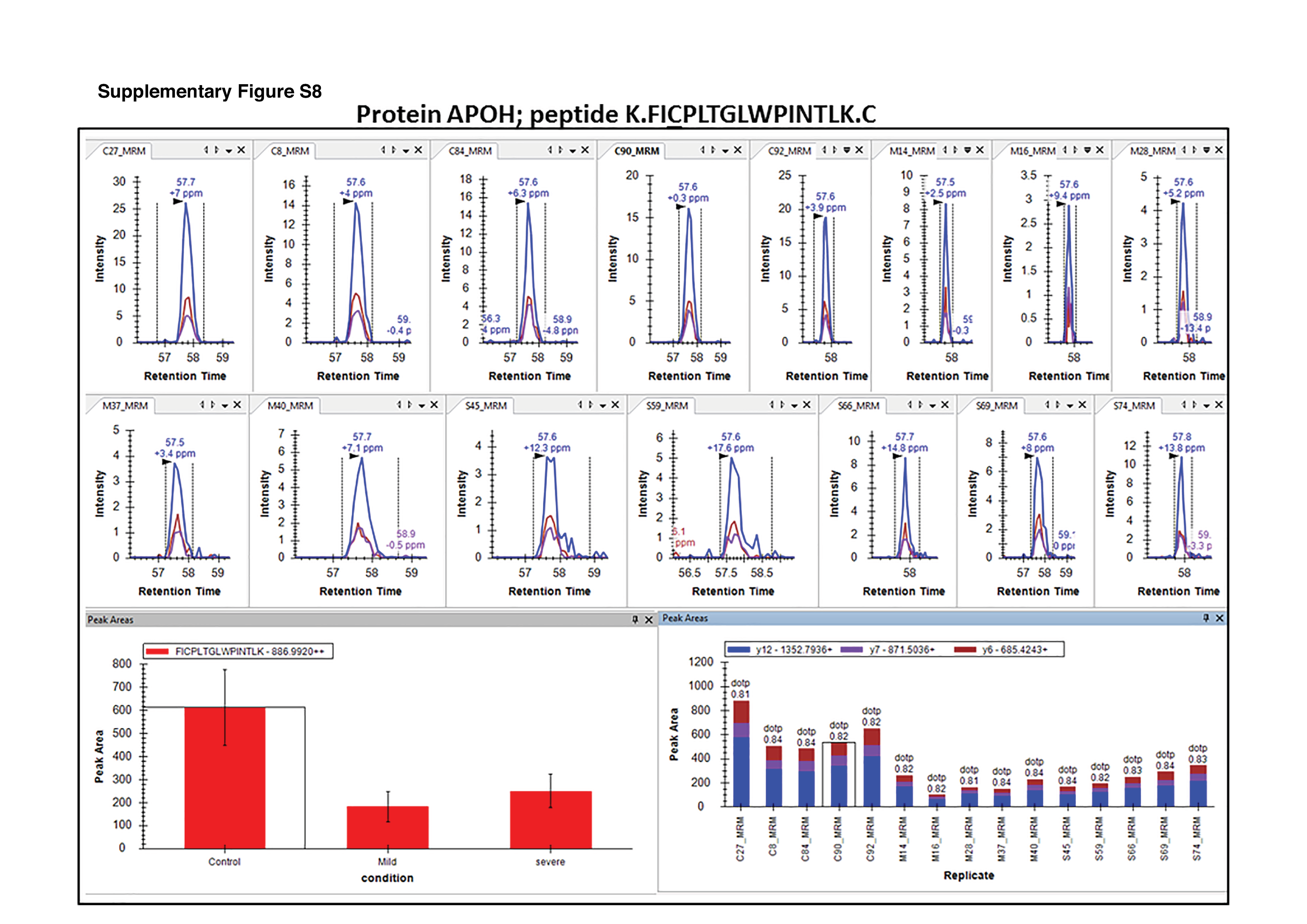

Supplement: Supplementary Figure S8 [file EMS176616-supplement-Supplementary_Figure_S8.tif]

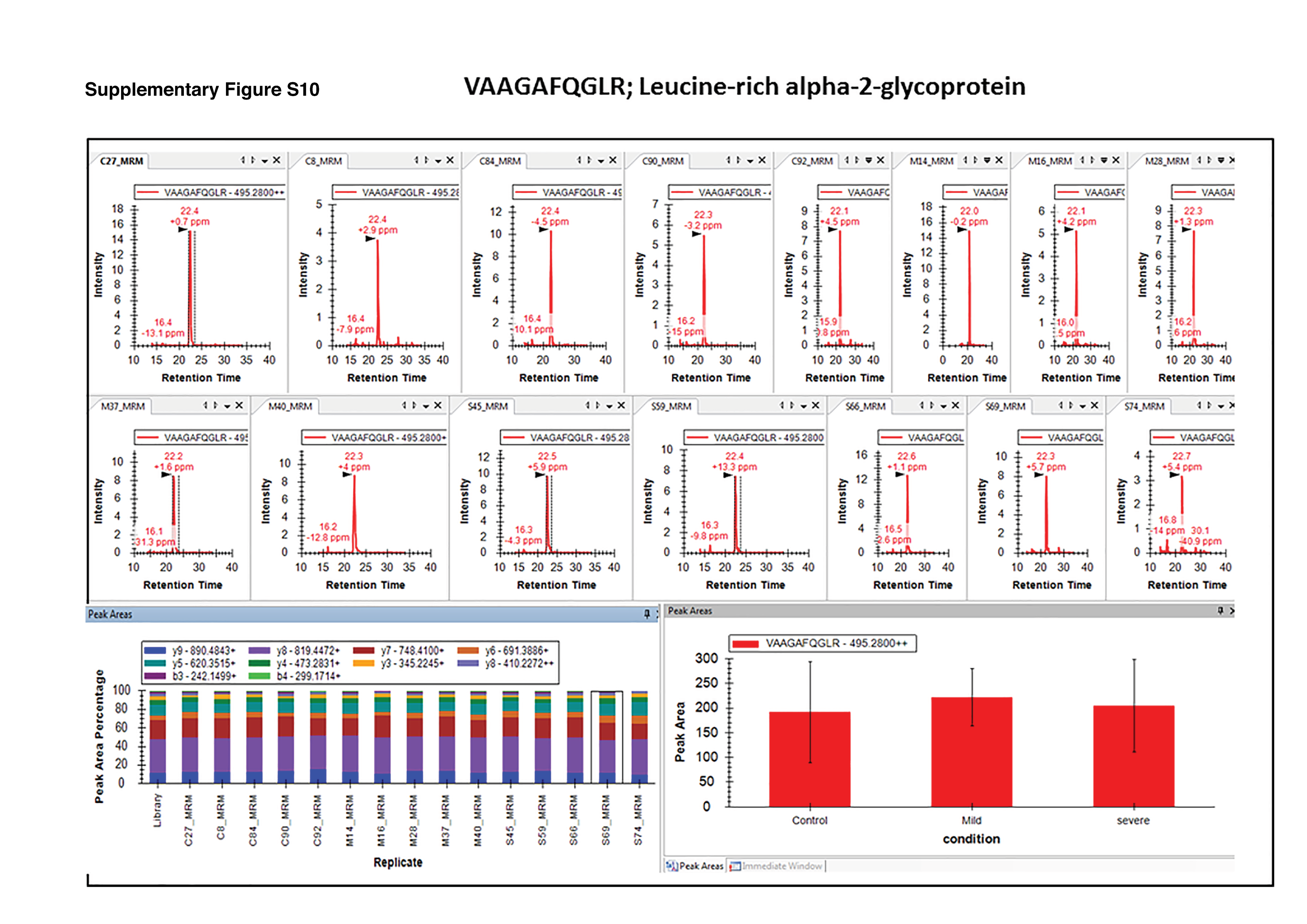

Supplement: Supplementary Figure S10 [file EMS176616-supplement-Supplementary_Figure_S10.tif]

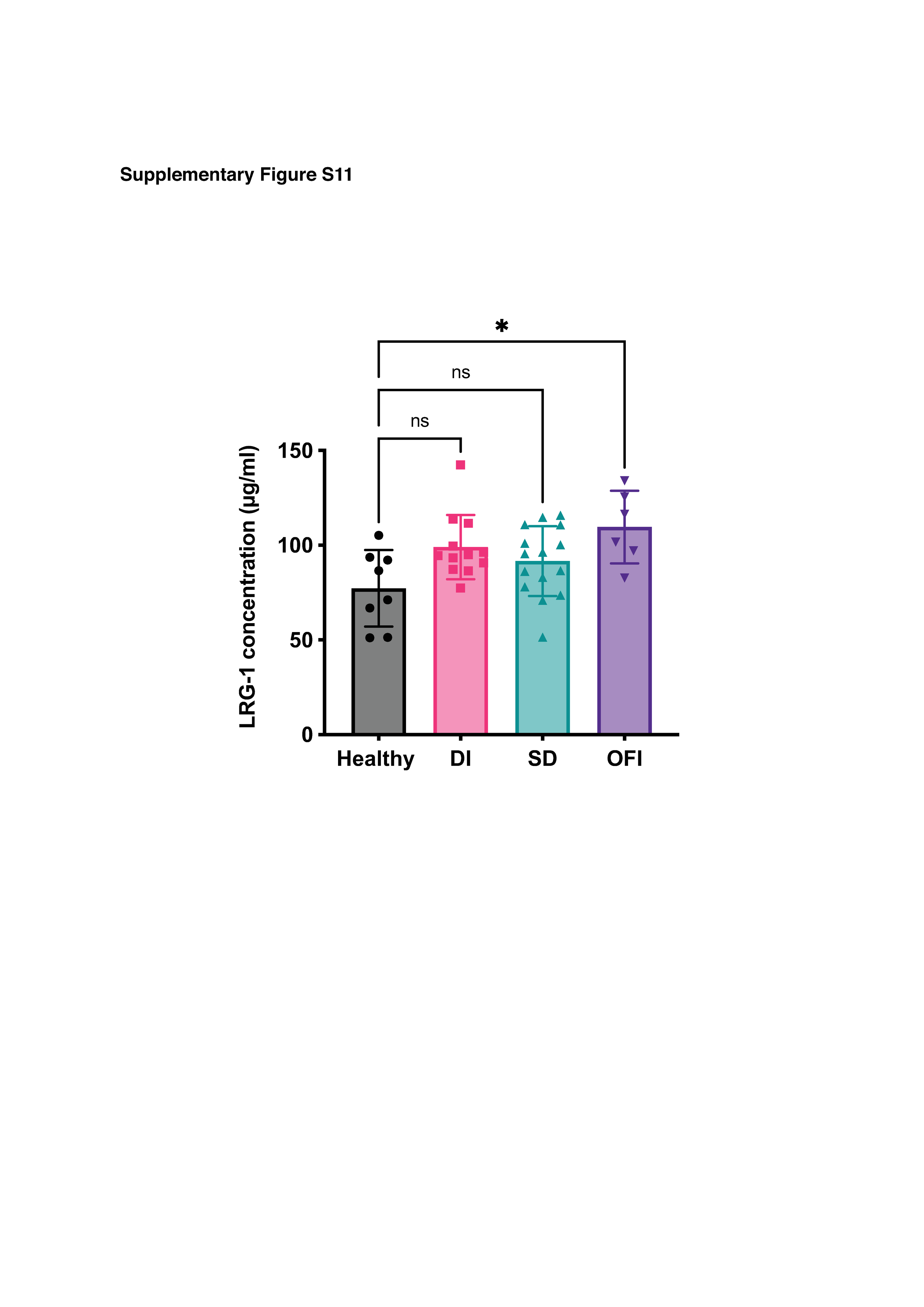

Supplement: Supplementary Figure S11 [file EMS176616-supplement-Supplementary_Figure_S11.tif]

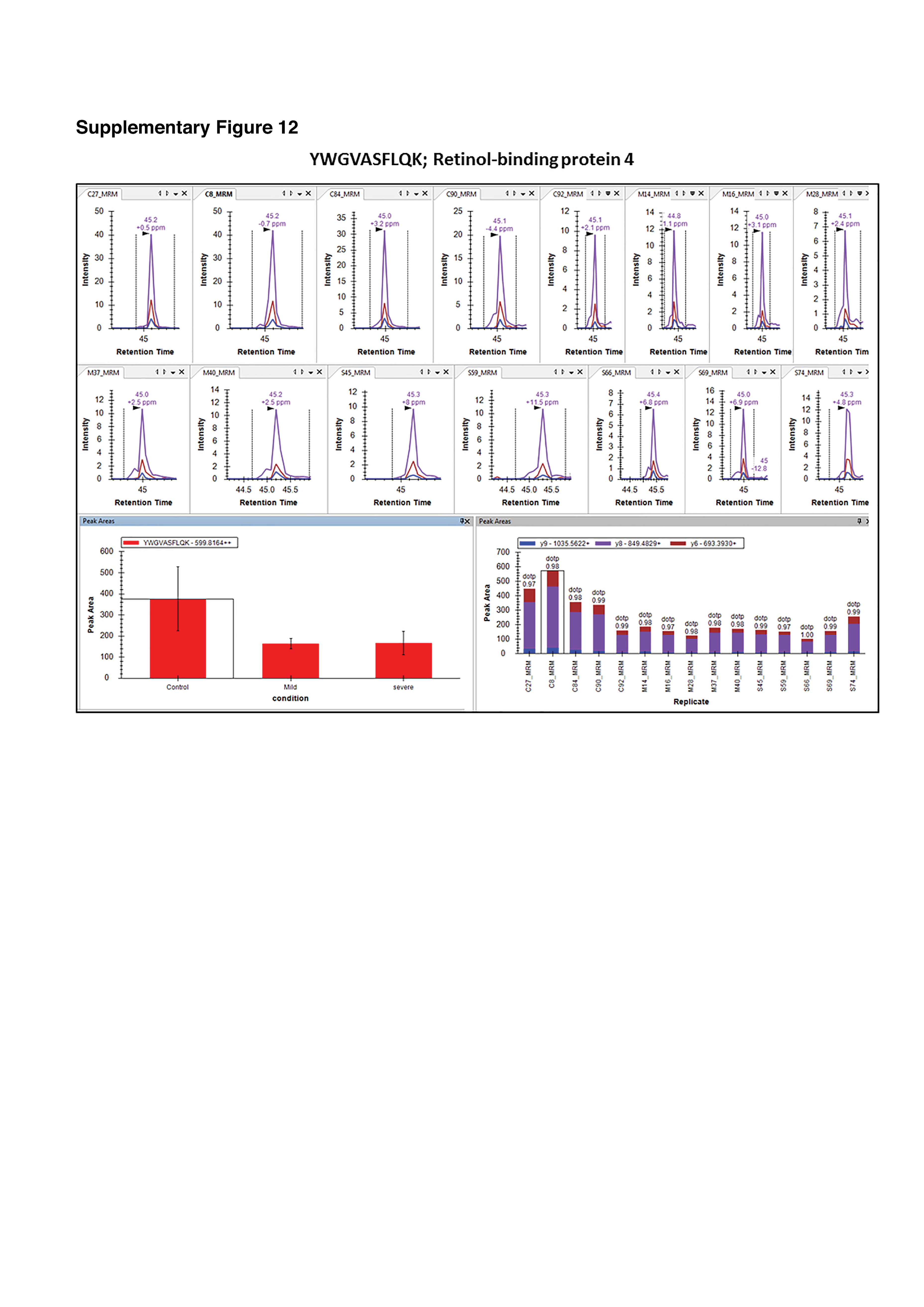

Supplement: Supplementary Figure S12 [file EMS176616-supplement-Supplementary_Figure_S12.tif]
