# Supplementary material for: Severe dengue in children associates with dysregulation of lipid homeostasis, complement cascade and retinol transport
Source: Clin Transl Med. Author manuscript; Available in PMC 2023 Jun 7. (PMC10230155; doi:10.1002/ctm2.1271)
Supplement: Supplementary Methods [file EMS176616-supplement-Supplementary_Methods.docx]

**SUPPLEMENTARY INFORMATION**

**METHODS**

All the key resources and reagents used in the study are listed at the end of this section.

**Patients enrolment and sampling**

Children aged 4-14 years, with less than five days of fever, attending All India Institute for Medical Sciences (AIIMS), Delhi, India who presented with clinical suspicion of dengue and confirmed using ‘Dengue Day 1 Test’ for NS1 antigen qualitative detection and IgM antibodies to DENV in serum/plasma were involved in the study. Written informed consent was obtained from patients before any study-specific procedures were performed. We categorized patients based on WHO classification into three groups: Dengue Illness (DI), Dengue with warning signs (DW), and Severe Dengue (SD). Patients were requested to come one month later for a follow-up and for the collection of convalescent samples. 4-5 ml blood sample was collected in Vacutainer CPT tubes, centrifuged at 700 x *g* for 20 min at room temperature (RT). Plasma was stored at -80 ºC in aliquots until analysis.

**Depletion of plasma abundant proteins**

Plasma samples were thawed on ice and centrifuged at 16,000 x g at 4 ºC for 5 min to pellet the precipitated material. 60 µg of plasma was used for depletion of abundant proteins using Pierce Top 12 Abundant Protein Depletion Spin Columns as per the manufacturer’s protocol. Depletion spin columns were equilibrated to room temperature (RT) followed by the addition of plasma to the resin slurry in the column. Columns were inverted several times to mix the resin in solution completely and incubated with gentle end-over-end mixing for 60 min at RT. After incubation, columns were placed in 2 ml collection tubes and centrifuged at 1000 x *g* for 2 min. The filtrate contains sample with depleted proteins.

**Protein precipitation, digestion and iTRAQ labeling**

Protein precipitation was done by adding three times the volume of the depleted plasma sample, of chilled acetone and kept overnight at -80 ºC. The samples were then centrifuged at 17,000 x *g* for 10 minutes and acetone was decanted. The pellet was washed with chilled acetone. The pellet was air-dried for 1 min and resuspended in dissolution buffer provided in the iTRAQ kit. Labeling of samples with iTRAQ reagents was carried out according to the manufacturer’s instructions. Digestion was performed by iTRAQ (4-plex) labeling protocol by AB Sciex. Briefly, the protein pellet was resuspended in dissolution buffer. 1 µl denaturant was added per sample (50 µg) and mixed vigorously to solubilize the protein. 2 µl of reducing agent was added and incubated at 60 ºC for one hour. After reduction, 1 µl cysteine blocking reagent was added and incubated at room temperature for 10 min. Trypsin was added in a ratio of 1:20 (Sequencing grade modified trypsin) and incubated at 37 ºC for 16 hours. iTRAQ reagents were dissolved in 70 µl ethanol, vortexed, and transferred to digested peptide samples. The labeling reaction was performed at room temperature for one hour before quenching by adding water. After labeling, samples were pooled into a new sample tube and vacuum-dried.

**Peptide fractionation and Mass spectrometry**

The pooled and concentrated sample after iTRAQ labelling was fractionated manually using SCX column (ICATTM Cartridge assembly kit). The sample was resuspended in 1 ml of loading buffer (10 mM KH_2_PO_4_, 25% (v/v) acetonitrile (ACN), pH adjusted to 3.0 using H_3_PO_4_) and loaded on the column using a syringe. The peptides were eluted using 1 ml elution buffer of increasing KCl concentration (10 mM KHPO_4_ pH 3, acetonitrile (ACN) (250 ml/L), and 30 mM, 50 mM, 70 mM, 100 mM 160 mM or 500 mM of KCl ) (pH adjusted to 3.0 using H_3_PO_4_). All the 1 ml fractions were speed vacuum dried and stored at -20 °C until running on mas spec. The samples were resuspended in solution buffer A (98 % water: 2% ACN) and subjected to LC-MALDI analysis. All fractions were analysed by 5600 Triple-TOF mass spectrometer which was connected to reverse-phase high-pressure liquid chromatography Ekspert nanoLC 415 system (Eksigent; Dublin, CA). Reverse phase elution was performed using trap elute function of Eksigent Nano LC with trap column (200 μm × 0.5 mm) and the C18 reverse phase analytical column (75 μm × 15 cm, 3 μm particle size C18). Mobile phase- A contained 0.1% formic acid in water, mobile phase-B 0.1% formic acid in acetonitrile. The flow rate during LC-run was set to 250 nL/minute to elute the peptides from the column with a gradient ranging from 90 % to 5 % eluent B. High-resolution TOF-MS scan over a mass range of 100–1250 m/z was used for the data-dependent acquisition (DDA) experiments followed by MS/MS scans of 25 ion candidates per cycle with activated rolling collision energy in high-sensitivity mode. The first selection criteria for the parent ions was the intensity and ions greater than 150 cps were considered. The charge state between +2 to +5 was considered as a second criterion. Positive-ion and high-sensitivity mode with a resolution of ~35,000 full-width half-maximum were used for mass spectra (MS) and tandem mass spectra (MS/MS). Collision-induced dissociation was triggered by rolling collision energy. The ion build-up time was set to 250 ms (MS) and to 70 ms (MS/MS).

**Protein identification**

Each MS run was performed twice to collect more data. Analyst TF 1.7 software was used to acquire the data. All MS run files were searched in Protein Pilot software v. 5.0.1 (SCIEX) with the Paragon algorithm for relative protein quantification and identification. Peptides were identified with Paragon algorithm search engine against Uniprot human protein database with *Homo sapiens* as the specified organism. The parameters for search included trypsin digested peptides with a maximum of one missed cleavage, iTRAQ-4plex, cysteine alkylation with methyl methanethiosulfonate (MMTS). Only peptides with a confidence score of > 0.05 were considered for further analysis and bias correction was automatically applied. False discovery rate analysis was also performed through decoy database. Oxidation of methionine (+15.995Da) was set as variable modification. Carbamidomethylation of cysteine (+57.021Da) and isobaric tags on N-termini on lysine (+145 Da) were used as static modifications. The peptide and product ion tolerance of 0.05 Da were used for searches. The output of this search is a group file that contains information such as protein name, accession, cleaved peptide sequence, modified peptide sequence, relative intensity, precursor charge, unused Protscore, confidence, and decoy result. Peptides from 12 abundant plasma proteins ( α1-Acid Glycoprotein, α1-antitrypsin,  α2-macroglubulin,  albumin, Apolipoprotein A-I, Apolipoprotein A-II, fibrinogen, haptoglobin, IgA, IgG, IgM, transferrin) were excluded from further analysis.

For filtering data following criteria were used: i) Unused score >0; ii) No decoys in accession (represented by RRRRR as prefix to protein names); iii) Peptides either >1 or >0; iv) Quantitative values present in 115:114, 116:114, 117:114 (no blank columns in p-value of each). The duplicate proteins with different protein group numbers but same quantitative values and peptide counts were removed from the analysis before combination. On a manual inspection, it was found that these proteins had duplicate entries. The proteins that passed 1% Global False discovery rate (G-FDR) and contained at least two unique peptides with 95% confidence were used for further analysis. Reporter ion ratio was used for the relative protein quantitation. For graphical representation and statistical analysis, convalescent samples were assigned an arbitrary value of around 1 for each of the five iTRAQ sets and compared as control samples.

**Retinol estimation**

100 µl serum sample was taken in a glass test tube, and 100 µl of retinyl acetate (100 µg/dl in ethanol) was added and vortexed for exactly 20 seconds using a cyclomixer. Then, the test tube was kept at 4 °C for 5 min. Then 1 ml hexane was added and vortexed exactly for one min. The mixture was then centrifuged for 10 min at room temperature at 2,500 rpm. After centrifugation, hexane layer was carefully transferred to another glass tube. Hexane was evaporated using nitrogen gas. After drying, 100 µl methanol was added, and the mixture was vortexed for 30 seconds. Finally, the sample was transferred into the autosampler vial and retinol levels were analyzed by HPLC (Shimadzu LC-6AD Binary Gradient System) using C-18 column (Supelco C-18, 10 microns) at a flow rate of 1.2 ml/min and under a pressure of 80-130 kgf. Methanol: water (95:5 v/v) was used as a mobile phase. Injection volume was 20 µl. Retinyl acetate was used as an internal standard. Retention time for retinol was 4.4 min and for retinyl acetate was 6.2min. UV-detector was used for detection at 326 nm of 1-10 min and 290 nm for 10-20 min. Retinol concentration was calculated by dividing the area of the retinol peak (in the sample) by the area of the retinol peak obtained separately by running the standard retinol alone at the fixed concentration of 100 μg/dl, and multiplying the result by 100. Correction factor (% recovery) was calculated by dividing the area of retinyl acetate peak (in the sample) by the area of retinyl acetate peak (obtained separately by running the standard retinyl acetate alone) and multiplying the result by 100. Results of the samples yielding 80% to 120% recovery value were included. Corrected retinol level was calculated by dividing the estimated retinol level by correction factor and multiplying the result by 100. In this manner, the values for retinol were adjusted for the recovery factor.

**High resolution multiple reaction monitoring (HR-MRM)**

We adopted HR-MRM technique to target specific peptides in the samples. 3 µl plasma sample was taken and diluted into 50 µl ammonium bicarbonate (ABC). Protein was estimated using Pierce BCA protein assay kit. Chilled acetone was added in a ratio of 1:3 followed by incubation at -80ºC for three hours for protein precipitation. After incubation, samples were centrifuged at 13,000 rpm for 15 min at 4 ºC. Acetone was removed completely and protein pellet was air dried. Pellet was resuspended in 500 mM triethylammonium bicarbonate (TEAB), vortexed briefly and sonicated for 30 min. Tris (2-carboxyethyl) phosphine (TCEP) was added at a final concentration of 5 mM followed by incubation at 50 ºC for 30 min. Iodoacetamide (IAA) was added to the solution to make a final concentration of 10 mM and incubated for 20 min at room temperature. Lys-C was added in a ratio of 1:50 and incubated at 37 °C for 4 hours followed by the addition of trypsin (1:20) for 16 hours at 37 °C. After digestion, formic acid (10%) was added at a final concentration of 0.1% to stop the reaction. Proteomics analysis was carried out on TripleTOF 5600 mass spectrometer. The gradient time program was set as follows: 0 to 2 min: 5%, 2 to 50 min: 10% to 30%, 50 to 60 min: 30% to 50%, 60 to 62 min: 50% to 90%, 62 to 71 min: 90%, 71 to 78 min: 5%. The HR-MRM acquisition consisted of one 200 ms TOF-MS scan from 350 to 1050 Da, followed by MS/MS scans from 100 to 1600 Da (90 ms accumulation time, 50 mDa mass tolerance, +2 to +5 charge states. operated in positive mode. The instrument settings parameters were as follows: gas1: 14 psi; gas2: 0 psi; curtain gas: 25 psi; ion spray voltage floating: 2100 V; turbo temperature: 130 ºC. Collision energies were calculated with the following formulae: CE = (m/z*0.044) +4 for +2 charge peptides and CE = (m/z*0.05) +3 for +3 charge peptides. The total cycle time, including 250 ms TOF-MS scan, was 4.62 s for all the peptides. The data generated was analyzed by Skyline which spontaneously identifies and matches the MS/MS chromatographic peaks against the spectral library created from the data-dependent acquisition (DDA) searches. All peaks shown by the software were checked manually after the automated matches to ensure the intensity distribution of selected transitions was consistent with the theoretical distributions in the spectral library. The fragments to be used for each peptide were chosen from the top three high-intensity fragments that had no apparent interference. The peak areas for peptides were calculated by summing the peak areas of their transition ions and proteins were quantified by the total peak area of their corresponding peptides. The fold change was determined by comparing the mean value of the peak areas of peptides among the samples. PeakView software (version 1.1.1.2 by Sciex) was employed to view the XICs for particular m/z values characteristic of different peptides. The occurrence of the peptide in the XIC profile was confirmed via a manual inspection of the MS/MS spectrum for the ion peaks identified by the mass spectrometer with the theoretical fragmentation spectrum for the peptide. Finally, the top three transitions were used from the MS/MS spectra and summed for peak area integration using skyline software.

The mass spectrometry proteomics data have been deposited to the ProteomeXchange Consortium via the PRIDE ^16^ partner repository with the dataset identifier PXD035690 and PXD018014.

**ELISA**

We analysed the presence of LRG1 in serum samples of patients with mild dengue (DI, n=12), severe dengue (SD, n=15), other febrile illness (OFI, n=5) and healthy control (n=8). The analysis was carried out by quantitative ELISA as per the manufacturer’s instructions. Assay was performed in duplicates (technical replicates) of each sample. Standard and diluted serum samples were added to microplate and incubated at room temperature. Wells were washed with buffer followed by addition of prepared biotinylated antibody into microplate to capture target protein. Following this, horseradish peroxidase-conjugated streptavidin was added. In the last step, 3,3,5,5'-tetramethylbenzidine (TMB) substrate was added followed by stop solution. Absorbance was measured at a wavelength of 450 nm using microplate reader. Unknown concentrations (ng/ml) of LRG1 were calculated by plotting standard curve and regression analysis. The graph was prepared by plotting the mean concentration of LRG1 on Y-axis and sample groups on X-axis. Final statistical analysis and graph for presentation was prepared using GraphPad prism software.

**Multiplex bead-based assay for Apolipoproteins**

The apolipoproteins were detected in the serum using Human Apolipoprotein (Apo) Panel (11-plex). The assay panel allowed the detection and quantitation of 11 different human Apo proteins including Apo AI, Apo AII, Apo B100, Apo CII, Apo CIII, Apo D, Apo E, Apo E4, Apo H, Apo J, and Apo M. This assay used fluorescence-encoded beads which were suitable for use on flow cytometers. Serum samples were diluted as per manufacturer’s protocol. Assay buffer was added to wells followed by the addition of standards and samples. Beads were loaded to the wells and incubated at room temperature. Wells were washed with wash buffer and detection antibodies were added. Following this, streptavidin-phycoerythrin conjugate was added and incubated. This was followed by beads resuspension in wash buffer. The signal intensity was measured using flow cytometry (BD FACS Canto). Absolute concentrations (mg/dl) of each analyte was determined by standard curve plotted using known amount of analyte provided in assay kit.

**Statistical tests**

Data was analysed and charts were prepared using GraphPad Prism software (version 9.5.1). Error bars represent mean ± SD. Statistical significance was estimated by *t*-test (unpaired, non-parametric). Multiquant software (SCIEX) was used for targeted quantitation of peptides identified by iTRAQ labeling. Statistical analysis of up and downregulated proteins from mass spec data was performed by Marker view (SCIEX). Normalization was performed using an internal standard (peptide transition of *E.coli* β-galactosidase). Statistical significance was estimated by non-parametric tests as indicated in figure legends using GraphPad Prism software.

**Table of reagents and resources used in the study**

| **REAGENTS OR RESOURCES** | **SOURCE** | **IDENTIFIER** |
| --- | --- | --- |
| **Biological samples** |  |  |
| Blood samples from dengue patients | All India Institute for Medical Sciences (AIIMS), New Delhi |  |
| **Kits and Reagents** |  |  |
| Dengue Day 1 test kit | J. Mitra and Co. Pvt. Ltd. | Catalog no: IR028050 |
| Panbio Dengue IgM capture ELISA kit | Abbott | Catalog no: 01PE10 |
| Panbio Dengue IgG capture ELISA kit | Abbott | Catalog no: 01PE20 |
| Pierce Top 12 Abundant Protein Depletion Spin Columns | Thermofisher Scientific | Catalog no: 85165 |
| iTRAQ reagents multiplex kit | AB Sciex | Catalog no: 4352135 |
| Pierce BCA protein assay kit | Thermofisher Scientific | Catalog no: 23225 |
| Human Apolipoprotein Apo Panel (11-plex) | BioLegend | Catalog no: 740453 |
| Human LRG1 ELISA kit | RayBiotech | Catalog no: ELH-LRG1 |
| Sequencing grade modified trypsin | Promega | Catalog no: V5111 |
| Iodoacetamide | Sigma-Aldrich | Catalog no: I5161 |
| Acetone | Merck | Catalog no: 67-64-1 |
| Lys-C, Mass Spec Grade | Promega | Catalog no: VA1170 |
| Ammonium bicarbonate | Sigma-Aldrich | Catalog no: A6141 |
| Triethylammonium bicarbonate (TEAB) | Sigma-Aldrich | Catalog no: T7408 |
| Tris (2-carboxyethyl) phosphine (TCEP) | Sigma-Aldrich | Catalog no: C4706 |
| Acetonitrile with Formic acid (LCMS grade) | Sigma-Aldrich/Merck | Catalog no: 900667 |
| Potassium chloride (KCl) | Sigma-Aldrich | Catalog no: P9541 |
| Formic acid | Sigma-Aldrich | Catalog no: 1.59002 |
| Water with 0.1% Formic Acid | Sigma-Aldrich/Merck | Catalog no.1.59013 |
|  |  |  |
| **Instruments and columns** |  |  |
| ICAT Cartridge assembly kit | SCIEX |  |
| 5600 Triple-TOF mass spectrometer | SCIEX |  |
| Ekspert nanoLC 415 system | Eksigent, Dublin, CA |  |
| C-18 columns; 10 microns | Supelco |  |
| LC-6AD Binary Gradient System | Shimadzu |  |
| Flow cytometer FACSCanto | Becton Dickinson |  |
|  |  |  |
| **Software and Algorithms** |  |  |
| PeakView software v.1.1.1.2 | SCIEX |  |
| Protein Pilot software v.5.0.1 | SCIEX |  |
| Prism (9.5.1) | GraphPad |  |
| Marker View | SCIEX |  |
| MultiQuant Software | SCIEX |  |
