# Supplementary material for: Severe dengue in children associates with dysregulation of lipid homeostasis, complement cascade and retinol transport
Source: Clin Transl Med. Author manuscript; Available in PMC 2023 Jun 7. (PMC10230155; doi:10.1002/ctm2.1271)
Supplement: Supplementary Table S1 and S2 [file EMS176616-supplement-Supplementary_Table_S1_and_S2.docx]

**Supplementary Table S1. Clinical and laboratory data of patients whose samples were used for iTRAQ labelling**

| **Variable** | **Convalescent**  **N= 5** | **Dengue Illness**  **N= 5** | **SD without leakage**  **N= 5** | **SD with**  **fluid leakage**  **N=5** | **p-value** | **Statistical**  **test** |
| --- | --- | --- | --- | --- | --- | --- |
| Platelet count at enrolment; (X 10^3^/ µL)^#^ | - | 39 (30,69) | 48 (42,60) | 45 (35,73) | 0.7705 | Kruskal-Wallis |
| Viremia (genome equivalents/ml blood)* | - | 38051 (55, 2.7 X 10^7^) | 28416 (1018, 7.9 x 10^5^) | 10965 (453,2.6 x 10^5^) | 0.7690 | Kruskal-Wallis |
| Day of fever^ | - | 5.2 (0.84) | 5.8 (1.1) | 6.6 (1.5) | 0.2367 | Kruskal-Wallis |
| NS1 antigen units^#^ | 5.5 (5.5,19.5) | 28.4 (19.2,194.3) | 17.6 (10.9, 428.8) | 20.6 (10.9, 169.2) | 0.8322 | Kruskal-Wallis |

**^#^** Median (Interquartile range)

**^*^** geometric mean (95% CI of the geo. mean)

**^^^** Mean (Std. Dev)

| **Peptide sequences** | **Protein names** | **Modified peptide sequences** | **Begin position** | **End position** | **Missed cleavage** | **Collision energy** |
| --- | --- | --- | --- | --- | --- | --- |
| VEIFYR | sp\|Q08380\|LG3BP_HUMAN | VEIFYR | 36 | 41 | 0 | 24.56 |
| TLEAQLTPR | sp\|P05546\|HEP2_HUMAN | TLEAQLTPR | 367 | 375 | 0 | 20.22 |
| LLDSLPSDTR | sp\|P05155\|IC1_HUMAN | LLDSLPSDTR | 276 | 285 | 0 | 22.67 |
| ADLFYDVEALDLESPK | sp\|P04196\|HRG_HUMAN | ADLFYDVEALDLESPK | 219 | 234 | 0 | 52.4 |
| VAAGAFQGLR | sp\|P02750\|A2GL_HUMAN | VAAGAFQGLR | 250 | 259 | 0 | 22.37 |
| FSGTWYAMAK | sp\|P02753\|RET4_HUMAN | FSGTWYAMAK | 37 | 46 | 0 | 32.31 |
| YWGVASFLQK | sp\|P02753\|RET4_HUMAN | YWGVASFLQK | 107 | 116 | 0 | 45.13 |
| FICPLTGLWPINTLK | sp\|P02749\|APOH_HUMAN | FIC[+57]PLTGLWPINTLK | 63 | 77 | 0 | 57.66 |
| ALVQQMEQLR | sp\|P06727\|APOA4_HUMAN | ALVQQMEQLR | 316 | 325 | 0 | 29.95 |
| SLAELGGHLDQQVEEFR | sp\|P06727\|APOA4_HUMAN | SLAELGGHLDQQVEEFR | 287 | 303 | 0 | 38.01 |

**Supplementary Table S2: List of peptides used in HR-MRM.**
